# Hypomethylation coordinates antagonistically with hypermethylation in cancer development: a case study of leukemia

**DOI:** 10.1186/s40246-016-0071-5

**Published:** 2016-07-25

**Authors:** Garima Kushwaha, Mikhail Dozmorov, Jonathan D. Wren, Jing Qiu, Huidong Shi, Dong Xu

**Affiliations:** 1 Christopher S. Bond Life Sciences Center, University of Missouri, Columbia, MO 65211 USA; 2 Informatics Institute, University of Missouri, Columbia, MO 65211 USA; 3 Department of Biostatistics, Virginia Commonwealth University, Richmond, VA 23225 USA; 4 Arthritis and Clinical Immunology Program, Oklahoma Medical Research Foundation, Oklahoma City, OK 73104 USA; 5 Department of Applied Economics & Statistics, University of Delaware, Newark, DE 19716 USA; 6 GRU Cancer Center, Georgia Regents University, Augusta, GA 30912 USA; 7 Department of Biochemistry and Molecular Biology, Georgia Regents University, Augusta, GA 30912 USA; 8 Department of Computer Science, University of Missouri, Columbia, MO 65211 USA

**Keywords:** Epigenetic regulation, DNA methylation, Hypomethylation, CLL, Cancer, Signaling pathway, 3′UTR, Enhancer

## Abstract

**Background:**

Methylation changes are frequent in cancers, but understanding how hyper- and hypomethylated region changes coordinate, associate with genomic features, and affect gene expression is needed to better understand their biological significance. The functional significance of hypermethylation is well studied, but that of hypomethylation remains limited. Here, with paired expression and methylation samples gathered from a patient/control cohort, we attempt to better characterize the gene expression and methylation changes that take place in cancer from B cell chronic lymphocyte leukemia (B-CLL) samples.

**Results:**

Across the dataset, we found that consistent differentially hypomethylated regions (C-DMRs) across samples were relatively few compared to the many poorly consistent hypo- and highly conserved hyper-DMRs. However, genes in the hypo-C-DMRs tended to be associated with functions antagonistic to those in the hyper-C-DMRs, like differentiation, cell-cycle regulation and proliferation, suggesting coordinated regulation of methylation changes. Hypo-C-DMRs in B-CLL were found enriched in key signaling pathways like B cell receptor and p53 pathways and genes/motifs essential for B lymphopoiesis. Hypo-C-DMRs tended to be proximal to genes with elevated expression in contrast to the transcription silencing-mechanism imposed by hypermethylation. Hypo-C-DMRs tended to be enriched in the regions of activating H4K4me1/2/3, H3K79me2, and H3K27ac histone modifications. In comparison, the polycomb repressive complex 2 (PRC2) signature, marked by *EZH2*, *SUZ12*, *CTCF* binding-sites, repressive H3K27me3 marks, and “repressed/poised promoter” states were associated with hyper-C-DMRs. Most hypo-C-DMRs were found in introns (36 %), 3′ untranslated regions (29 %), and intergenic regions (24 %). Many of these genic regions also overlapped with enhancers. The methylation of CpGs from 3′UTR exons was found to have weak but positive correlation with gene expression. In contrast, methylation in the 5′UTR was negatively correlated with expression. To better characterize the overlap between methylation and expression changes, we identified correlation modules that associate with “apoptosis” and “leukocyte activation”.

**Conclusions:**

Despite clinical heterogeneity in disease presentation, a number of methylation changes, both hypo and hyper, appear to be common in B-CLL. Hypomethylation appears to play an active, targeted, and complementary role in cancer progression, and it interplays with hypermethylation in a coordinated fashion in the cancer process.

**Electronic supplementary material:**

The online version of this article (doi:10.1186/s40246-016-0071-5) contains supplementary material, which is available to authorized users.

## Background

Loss of DNA methylation, also known as hypomethylation, in cancer cells relative to normal cells was one of the first-described epigenetic changes in human cancers. Hypomethylation has been detected at both a global level and on a local scale [1] in cancer genomes. Many cancer types have been reported to have global loss of methylation like glioblastoma [2], ovarian epithelial carcinoma [3], prostate metastatic tumors [4], B cell chronic lymphocytic leukemia [5, 6], hepatocellular carcinoma [7], cervical cancer [8], colon adenocarcinoma [9], and Wilms’ tumor [10]. However, the biological significance of DNA hypomethylation remains understudied owning to its unclear role in carcinogenesis, in contrast to hypermethylation, which is commonly viewed as a transcription silencing mechanism [11, 12]. Yet, hypomethylation of DNA, despite its unclear role, has been linked to tumor progression [8, 13] in different tumor types and in individual specimens [3, 14]. Also, some experiments have indicated the importance of induced DNA hypomethylation in oncogenesis by using DNA methylation inhibitors in vivo and in vitro [15, 16]. However, the role of hypomethylation is not clearly understood. Hence, it is critical to analyze hypomethylation data in depth to achieve a better understanding of its biological roles in carcinogenesis.

DNA hypomethylation in cancer is often seen in satellite DNAs, Arthrobacter luteus (ALU) repeats, and long interspersed nuclear elements (LINEs) [17, 18], etc. These DNA repeats comprise approximately half of the genome. Hence, DNA hypomethylation is generally considered a global phenomenon not suitable for use as a biomarker. One advantage of the global hypomethylation phenomenon (as it pertains to its genome composition) is that it is often considered a technique to balance focal and conserved hypermethylation in the promoter regions of key genes. Also, it is believed that these hypomethylated genomic regions are randomly spread over the genome, mostly in repetitive regions whose functions, if any, are unclear. Again, this reported disadvantage might actually be an advantage due to recent findings indicating that ALU elements can act as enhancers [19], which further emphasizes the need for defining the role of hypomethylation in cancers.

As part of our study of hypomethylation patterns, we used B cell chronic lymphocytic leukemia (B-CLL) as an example case. This B-CLL cancer type has a predominant global hypomethylation as its characteristic feature [5, 6], and it is the most common form of blood cancer. It is a clinically heterogeneous disease, with some patients experiencing rapid disease progression and others living for decades without requiring treatment [20]. Although a number of cellular and molecular prognostic markers, i.e., surface markers *ZAP70* and *CD38*, cytogenetic abnormalities, and IGHV mutational status [21–23], have been identified to help classify CLL into molecular and clinical subgroups and to predict their course of progression, they do not provide clear insight into the underlying biology necessary to develop better targeted and more effective treatments.

In addition to various molecular and genetic changes, several genome-wide DNA methylation studies have identified many aberrantly methylated genes in CLL samples [24]. Initially, DNA hypermethylation in CLL patients was found to affect 4.8 % of CpG islands on average [25]. Furthermore, hypermethylation in the promoters of tumor suppressor genes such as *DAPK1* [26], *SFRP1* [27], and *ID4* [28] genes involved in apoptosis, cell cycle regulators *p16* and *p15* [29], and prognostic markers *ZAP70* [21] and *TWIST2* [30] were identified. DNA methylation changes were also found to be associated with disease progression in the E_μ_-TCL1 transgenic mouse model of CLL [28]. In addition to hypermethylation, hypomethylation of proto-oncogenes has also been observed particularly in liver tumors and leukemia such as the *c-fos*, *c-myc*, *ras*, *Erb-A1* [31], and the *bcl-2* gene [32]. Along with this, many studies have indicated widespread hypomethylation compared to instances of hypermethylation, particularly in the CLL cancer type. However, a detailed account on the genome-wide hypomethylation pattern and its contributing role towards cancer development has not been conducted for CLL. Hence, it is clear that an in-depth methylation analysis focusing more on hypomethylation can be very helpful to unveil the underlying mechanism regulating the disease.

Here, we studied the genome-wide DNA methylation pattern in CLL and investigated whether hypomethylation is also consistent at some locations like hypermethylation across multiple CLL patients. We also investigated the biological role of consistent hypomethylation towards tumor initiation and progression; and finally, we compared instances of consistent hypomethylation to that of consistent hypermethylation. We characterized the epigenetic context of hyper- and hypomethylated regions in CLL and further investigated association of hypomethylation with change in expression of the neighborhood genes along with their potential mechanism of influence.

## Results

### Methylation data analysis

In order to study genome-wide methylation changes in the CLL genome, we computed differentially methylated regions (DMRs) from genome-wide methylation data of 30 samples from publically available CLL samples in GEO (http://www.ncbi.nlm.nih.gov/geo/). DMRs of size 1000 bp were obtained by comparing each patient sample against each control normal sample individually using Fisher’s exact test. False discovery rate (FDR) was used to correct for multiple testing errors with a *q* value threshold of 0.01. Hence, three sets of DMRs were obtained by comparing all 30 CLL samples against each of the three control samples.

#### Entropy and permutation analysis

**Fig. 1:**
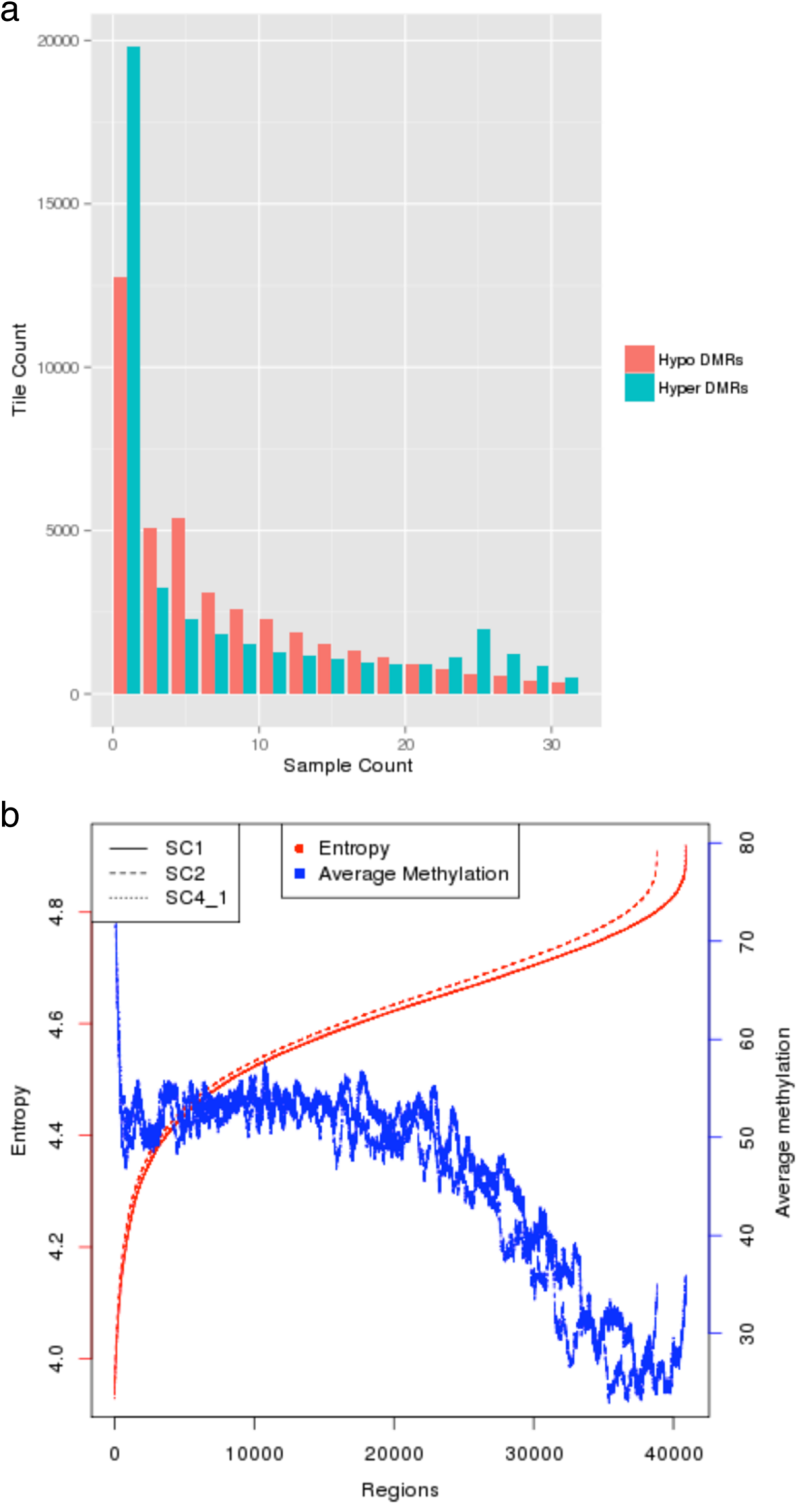
Overall representation of methylation. **a** Distribution of hyper/hypo-DMRs (tiles) over number of samples. This illustration shows that a higher proportion of hypo-DMRs (compared to hyper-DMRs) are consistent in small subsets of samples. It also shows the presence of few hypo-DMRs present in all 30 samples. **b** Relationship between average methylation difference across all CLL patients against control samples (SC1, SC2, and SC4_1) and methylation entropy per 1000 bp region

Having obtained a list of all hypo- and hyper-DMRs for each CLL against each control sample, we first plotted the distribution of the number of samples in which each DMR (hypo and hyper) existed. Figure 1a shows that the majority of hypo-DMRs are present in less than 50 % of samples. Out of the DMRs present in 20 or more samples, hyper-DMRs outnumbered hypo-DMRs. This showed that overall hypomethylated regions are less conserved than hypermethylated regions across samples.

Next, in order to check randomness in contrast to conservation of methylation change in each 1000 bp region across all CLL samples, methylation entropies were calculated using a probability distribution of methylation changes for each region. Figure 1b shows this opposite pattern of entropy and average methylation change. This plot shows that a high percentage methylation of specific regions is more consistent across all patients; however, as the average methylation goes down, their conservation tends to fluctuate, thereby leading to an increase in entropy (Fig. 1b). After comparing these methylation entropies for each region against the average methylation change across 30 CLL samples, we observed a negative correlation (Pearson correlation = −0.22, *p* value <0.05) in each of the 3-control sample tests.

#### Identification of consistent differentially methylated regions (C-DMRs)

In order to obtain consistent DMRs, a binomial test was used to check the significance of each DMR with the probability of being hypo/hypermethylated in 25 or more CLL samples (*q* value <0.01). Hence, three lists of significant DMRs were obtained for each control sample. Next, 658 hypo- and 982 hypermethylated regions that were found common in all three lists and referred to as C-DMRs (see Tables S1–S4 in Additional file 1 and Additional file 2 for lists and details).

To further check the statistical significance of our lists of hypo- and hyper-C-DMRs, we performed two permutation tests, one by permuting samples and the other by permuting methylation values for 1000 bp regions (see Methods in Additional file 1 for more details). The sample permutation test helped in detecting whether we observed hyper/hypo-C-DMR patterns by chance if there was no difference among cancer vs. normal samples. On the other hand, the methylated region permutation test detected whether hyper/hypo-C-DMR pattern can occur by chance if there is no difference between regions. In both permutation tests, all of our obtained C-DMRs had *q* values <0.05, showing the statistical significance of hyper- and hypo-C-DMRs in cancer samples against normal and non-DMRs.

#### Differences in positional genomic location analysis of hyper- and hypo-C-DMRs

**Fig. 2:**
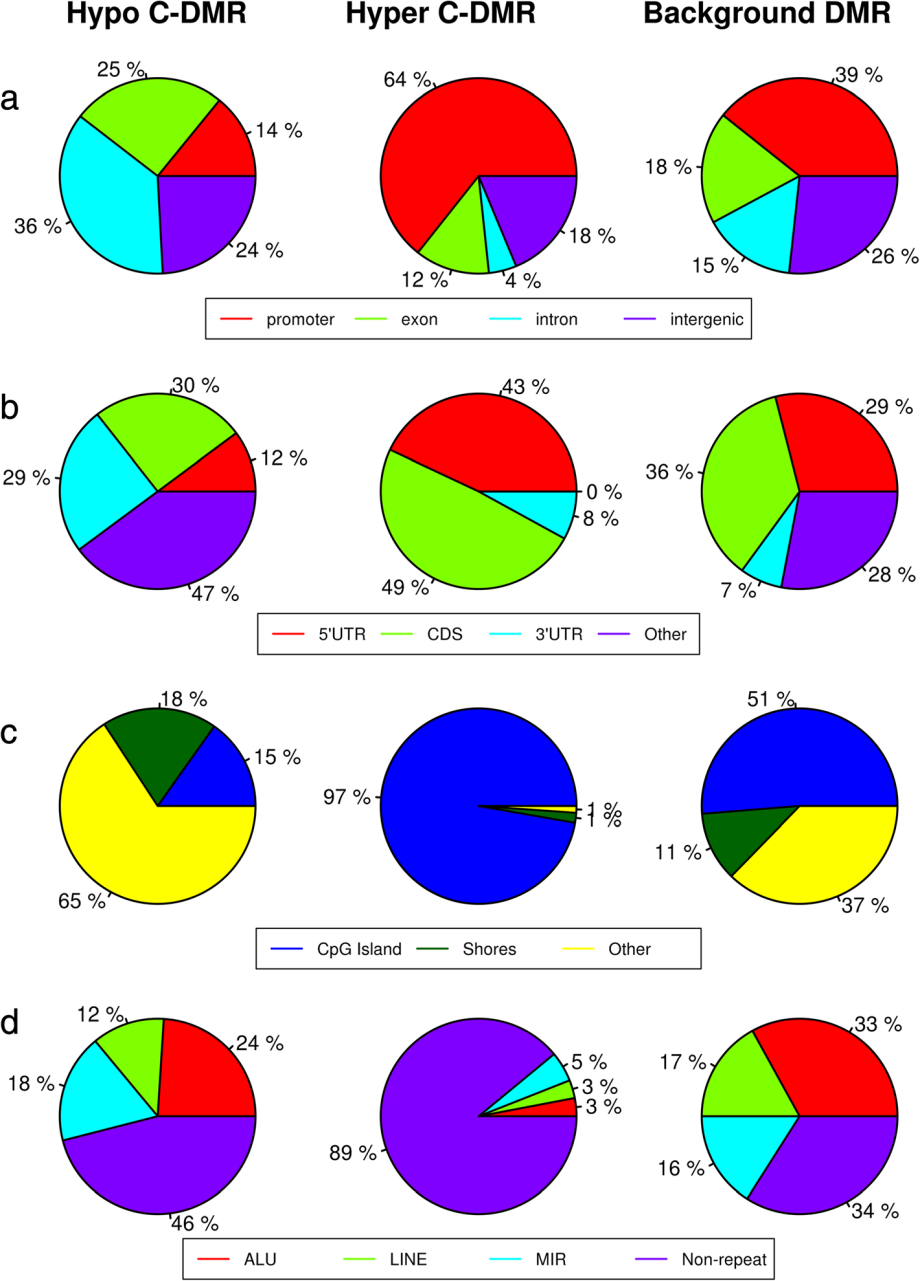
Distributions of C-DMRs. **a**, **b** Over different genic parts. **c** Over CpG islands. **d** Over genomic repeats and non-repeat regions

By checking the genomic-location distribution of C-DMRs, we found that a higher number of hyper-C-DMRs mapped to promoters (64 %) and 5′UTRs (43 %) as compared to hypo-C-DMRs (14 % for promoters and 12 % for 5′UTR) and genomic background regions (30 % for promoters and 29 % for 5′UTRs). A higher percentage of promoter and 5′UTR hypermethylation confirmed their role in interfering with transcription factor binding (Fig. 2a, b). However, hypo-C-DMRs outnumbered both hyper-C-DMRs and background genomic regions for 3′UTRs (29 % in hypo-C-DMRs, 8 % in hyper-C-DMRs, and 7 % in background) and introns (36 % in hypo-C-DMRs, 4 % in hyper-C-DMRs, and 15 % in background). There were also more hypo-C-DMRs (24 %) in the intergenic regions than hyper-C-DMRs (18 %), but comparable with genomic background regions (26 %). CpG sites from hypo- and hyper-C-DMRs were also checked against “weak/strong enhancer regions” chromatin states as defined by chromHMM [33] and were found coming mostly from intronic and intergenic regions on a genome. (Figure S6 in Additional file 1). Strong enhancers were overlapped more by hypo-C-DMRs in comparison to hyper-C-DMRs. Genes overlapping with hypomethylated strong enhancers were *ELN*, *GTF2I*, *KLC1*, *MIF4GD*, *MIR6821*, *MOB2*, *PTBP1*, *RGS3*, *SH3BGRL3*, *TBC1D14*, *TCIRG1*, and *VASH2*.

Across each sample data, we found 25–30 % of all DMRs as hypomethylated, and only 10–15 % hypermethylated, as shown in Figure S1 in Additional file 1. However, they were not targeted for any specific chromosomal region (Figure S2 in Additional file 1). Also, only 100 hypo-C-DMRs (15.2 %) co-localized with CpG islands, while 955 out of 982 hyper-C-DMRs (97.2 %) co-localized with CpG islands. Hypo-C-DMRs were mostly present in regions outside CpG islands and shores (Fig. 2c). Next, Fig. 2d shows that almost half of hypo-C-DMRs (46 %) were present on non-repeat regions along with ones mapped on the repeat regions. Overall, hypo-C-DMRs were found more in 3′UTR, intronic, and intergenic regions, mostly outside CpG islands and overlapping both repeat and non-repeat regions over enhancers. Additional file 3 lists the genes with genic-regions overlapped by these C-DMRs.

#### Enrichment of KEGG pathways, GO annotations and phenotypes

**Fig. 3:**
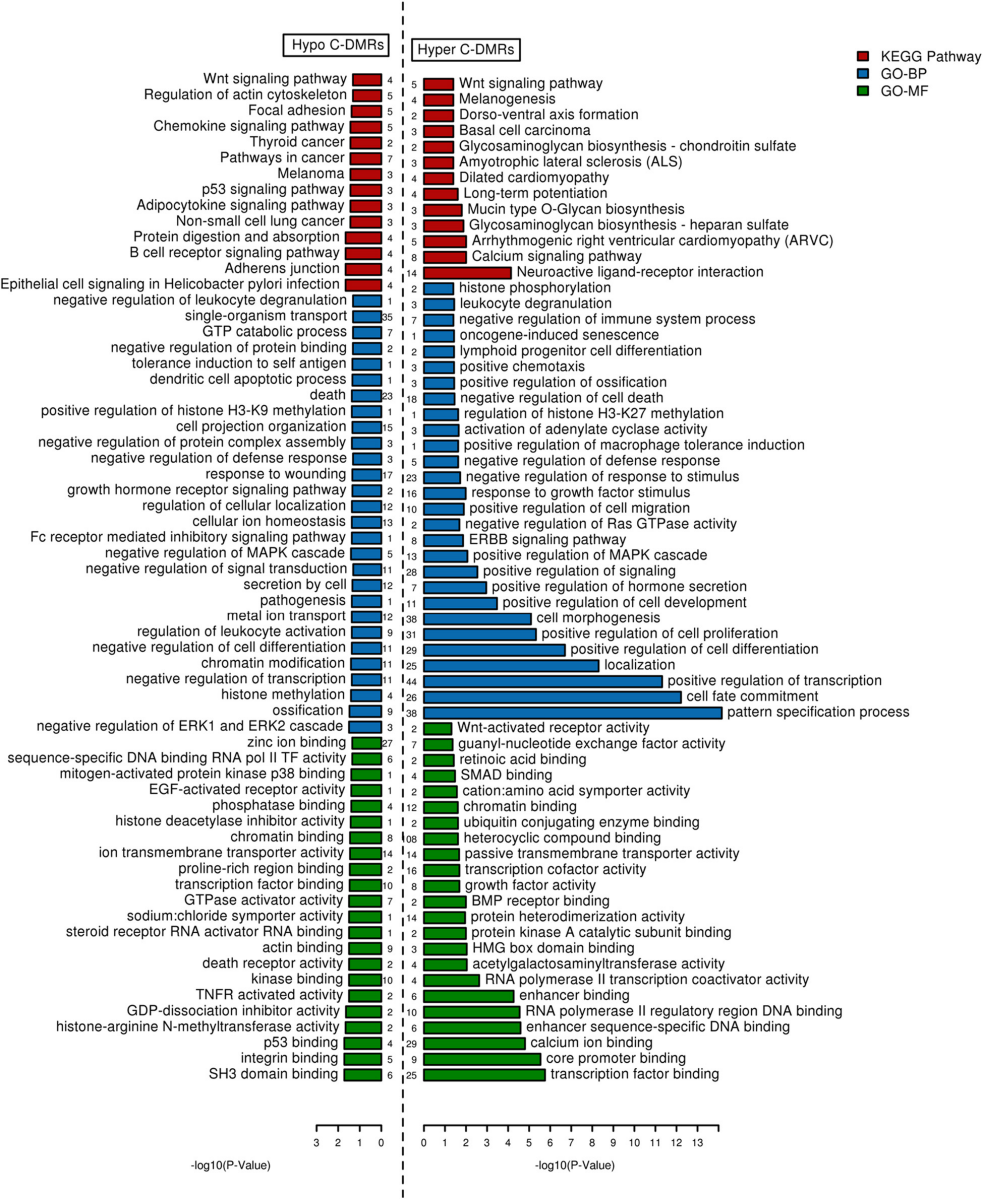
GO annotation and KEGG pathway enrichment for C-DMRs

For our obtained lists of hypo- and hyper-C-DMRs, we performed enrichment analysis of genes overlapped by C-DMRs (Additional file 3) for gene ontology (GO) [34] categories and KEGG [35] pathways. The most significantly enriched KEGG pathways in hypo-C-DMRs were “B cell receptor (BCR) signaling pathway” (adjusted *p* value (p.adj) = 2.21E-02), “*p53* signaling pathway” (p.adj = 3.69E-02), and “pathways in cancer” (p.adj = 3.69E-02), along with a few other signaling pathways and pathways involved in cancer. In contrast, hyper-C-DMRs were not enriched for any leukemia-related pathway such as *BCR* signaling. Hyper-C-DMRs were enriched for “neuroactive ligand-receptor interaction” (p.adj = 7.29E-05) and for the “calcium-signaling pathway” (p.adj = 9.86E-03) (Fig. 3). See Additional file 4 for complete enrichment results.

Among the GO annotations, the most important and significantly enriched biological processes for hypo-C-DMRs were “negative regulation of transcription” (p.adj = 4.28E-02), “chromatin modification” (p.adj = 3.92E-02), “regulation of signaling” (p.adj = 3.92E-02), “histone methylation” (p.adj = 3.92E-02); “positive regulation of histone H3-K9 methylation" (p.adj = 4.29E-02), “protein alkylation” (p.adj = 4.28E-02), “programmed cell death” (p.adj = 4.30E-02), “negative regulation of cell proliferation” (p.adj = 4.40E-02), and “negative regulation of leukocyte differentiation” (p.adj = 3.04E-02), “leukocyte activation” (p.adj = 4.28E-02), and “cell morphogenesis involved in differentiation” (p.adj = 4.29E-02). On the other hand, hyper-C-DMRs were enriched for processes which are antagonistic to the processes enriched in hypo-C-DMRs. Hyper-C-DMRs were enriched for processes like “positive regulation of transcription” (p.adj = 4.81E-12) and “positive regulation of cell differentiation” (p.adj = 1.99E-07), “positive regulation cell proliferation” (p.adj = 4.75E-06), “regulation of signaling” (p.adj = 3.53E-05) with more processes related to “cell-fate commitment” (p.adj = 6.09E-13), “cell morphogenesis involved in differentiation” (p.adj = 7.69E-08), and similar processes like “protein and cell localization” (p.adj = 4.77E-06). Also, hypo-C-DMRs were enriched for “H3K9 methylation” but hyper for “H3-K27 methylation.”

Molecular functions (Fig. 3) showed a strong enrichment for binding functions like “protein binding” (like integrin, p.adj = 1.92E-02), “SH3 domain binding” (p.adj = 1.92E-02), “p53 binding” (p.adj = 1.92E-02), “histone-arginine *N*-methyltransferase activity” (p.adj = 2.20E-02), “tumor necrosis factor-activated receptor activity” (p.adj = 3.20E-02), GTPase regulator activity (p.adj = 3.20E-02), and “transcription factor binding” (p.adj = 3.40E-02) in hypo-C-DMRs. In comparison, hyper-C-DMRs were specifically highly enriched for “sequence-specific DNA binding” (p.adj = 5.49E-32), transcription factor (or regulator, p.adj = 3.86E-25), and trans-membrane transporter activity (p.adj = 6.58E-03), along with also metal/ion (calcium, p.adj = 6.72E-07) and “enhancer binding” (p.adj = 5.47E-05). In summary, both C-DMR types (hypo and hyper) affected similar processes but in different directions, with hypomethylation focusing on transcription and leukocyte activation and hypermethylation focusing on transcription suppression through transcription factors and cell-fate commitment.

We did not observe any differences in enriched phenotypes between hypo- and hyper-C-DMRs. Specific associations with the hematopoietic system, homeostasis/metabolism, mortality/aging, immune system, and growth/size phenotype were enriched in both types of C-DMRs, except embryogenesis (or related terms like differentiation), which was specific to hypermethylation. Genes falling under each enriched top attribute are listed in Additional file 5. These results suggest the importance of both hypo- and hyper-C-DMRs in CLL, and emphasize the functional significance of hypo-C-DMRs.

#### Enrichment of TFBS, histone modifications, and chromatin states

Next, in order to identify epigenomic signatures associated with both C-DMRs, we systematically tested for enrichment in transcription factor binding sites (TFBSs), histone modification marks, and chromatin states provided by ENCODE project [36].

**Fig. 4:**
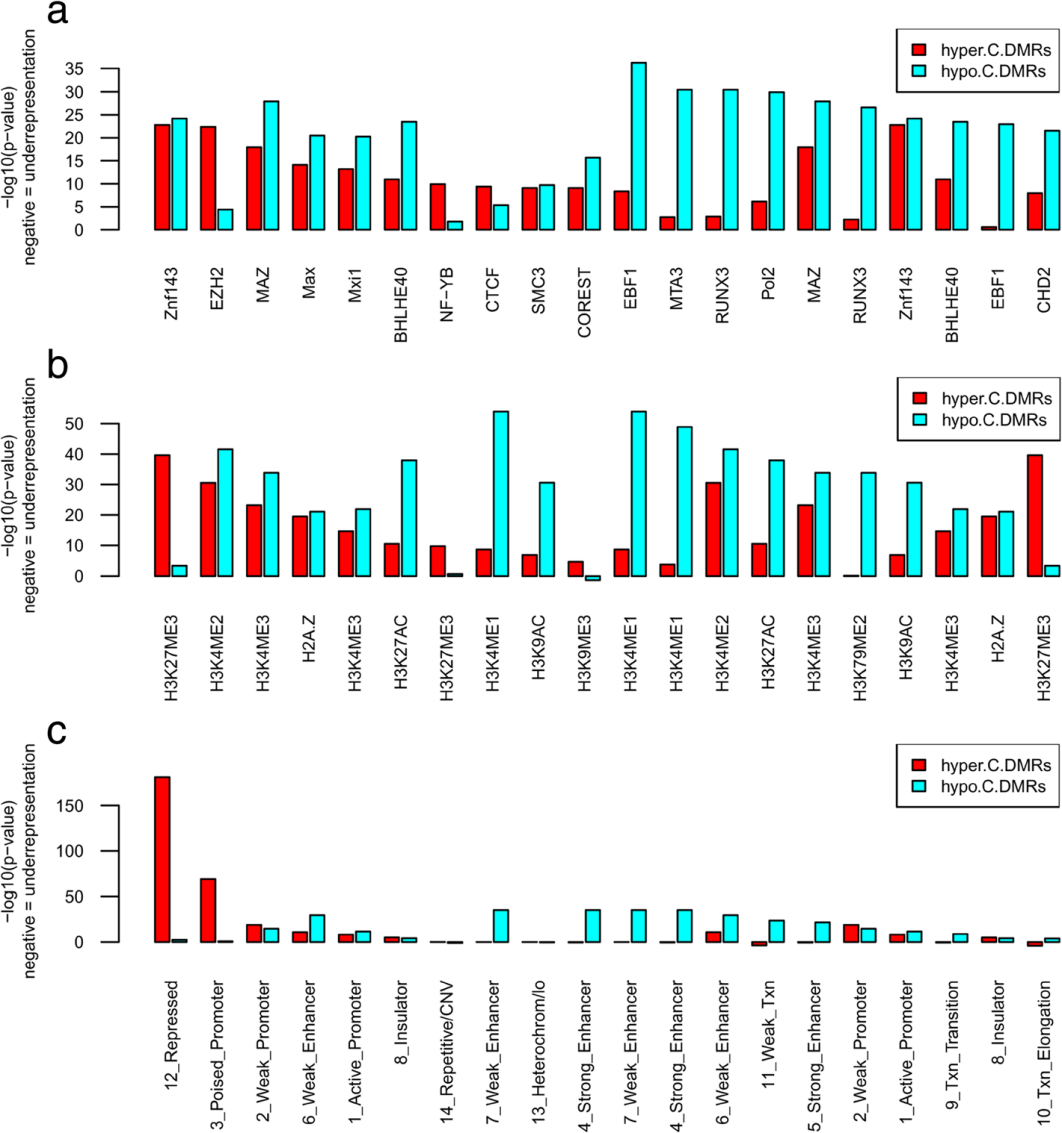
Enrichment of C-DMRs in the regulatory regions. The ENCODE genome annotation datasets for Gm12878 lymphoblastoid cell line from Broad and Stanford/Yale/USC/Harvard were used for transcription factor and Histone enrichment analyses (**a**, **b**). Broad datasets were used for chromatin states enrichment (**c**). See Figures S3, S4 in Additional file 1 for the results for all cell lines. The vertical axis shows –log10-transformed enrichment *p* values, FDR corrected. Left/right parts of the *bar*-*plots* show top 10 most significant enrichments for hyper/hypo-C-DMRs, respectively. **a** Transcription factor binding sites enrichment. **b** Histone modification sites enrichment. **c** Chromatin states enrichment

TFBS enrichment analysis identified EZH2 as strongly associated with hyper-C-DMRs (p.adj = 1.70E-23), in contrast to hypo-C-DMRs (p.adj = 4.27E-05, Fig. 4a). On the contrary, *EBF1*, *POL2*, *CHD2*, *WHIP*, and *TBLR1* were strongly associated with hypo-C-DMRs (p.adj = 2.53E-37, 1.06E-30, 2.17E-22, 6.024E-22, and 5.78E-19, respectively), in contrast to hyper-C-DMRs (p.adj = 1.65E-09 and 3.50E-07) (Fig. 4). Other than these, the HAIB dataset in ENCODE showed additional B lymphopoiesis-related enriched TFBS like *RUNX3* (p.adj = 3.58E-31), *TCF3* (p.adj = 4.04E-14), *PU.1* (p.adj = 7.42E-11), and *PAX5* (p.adj = 7.43E-11). Both hyper- and hypo-C-DMRs were enriched in *ZNF143*, *CTCF*, *MAZ*, and *MXI1* TFBSs (Fig. 4a). These findings were confirmed by using TFBS data from all cell lines provided by the ENCODE project (Figure S3 (a), S4 in Additional file 1 and Additional file 6).

Among histone modification marks, we found H3K27me3 to be highly enriched in hyper-C-DMRs (Fig. 4b, p.adj = 2.31E-40). On the contrary, hypo-C-DMRs were strongly enriched in H3K4me1 (p.adj = 1.11E-54), H3K27ac (p.adj = 1.22E-38), and H3K79me2 (p.adj = 1.44E-34) histone modification marks (Fig. 4b). Both C-DMRs were enriched in H3K4me2, H3K4me3, and H2AZ histone modification marks (Fig. 4a). The specificity of the H3K27me3 mark for hyper-C-DMRs was confirmed by using histone modification data from all cell lines provided by the ENCODE project (Figure S3 (b) in Additional file 1 and Additional file 6).

Furthermore, hyper-C-DMRs were significantly enriched for homeobox, *E2F* and TATAbox/promoter motifs, and hypo-C-DMRS for *ETS*, *IRF4*, *EGR*, *ZFX*, *RUNX1*, *PU.1*, *Pax5*, *BATF*, *Erra*, and *bZIP* motifs (Table S5 in Additional file 1). All motifs enriched for hypo-C-DMR have been shown to contribute to cell proliferation, B cell development and pathogenesis of lymphomas [37–39].

Using chromatin state annotations from multiple cell lines (Figure S3 (c) in Additional file 1 and Additional file 6), we found hyper-C-DMRs to be enriched in “repressed” (p.adj = 5.63E-182) and “poised promoters” (p.adj = 1.24E-69) chromatin states (Fig. 4c). In contrast, hypo-C-DMRs were consistently enriched in both “strong/weak enhancers” (p.adj = 6.73E-36 for both) and “weak transcription” (p.adj = 2.73E-24). Both C-DMRs were similarly enriched in “weak promoters” (p.adj = 1.70E-19 and 2.93E-15 for hyper- and hypo-C-DMRs, respectively). These results suggest that distant enhancer regions tend to be hypomethylated in cancer whereas regions associated with repressed or poised transcription are hypermethylated.

Also, 17,811 hyper- and 15,599 hypo-differentially methylated regions (DMRs) were obtained from pooled CLL and control sample comparison (Table S2 in Additional file 1). Each of these region lists exhaustively included all hyper- and hypo-C-DMRs, respectively. KEGG and GO annotation enrichment analysis of pooled sample DMRs also showed a strong enrichment of similar terms (Additional files 1 and 4). Results from pooled sample analysis show that C-DMRs are more specific and significant subset of DMRs that are consistently present in all samples.

### Expression data analysis

#### Expression profiles in relation with methylation

**Fig. 5:**
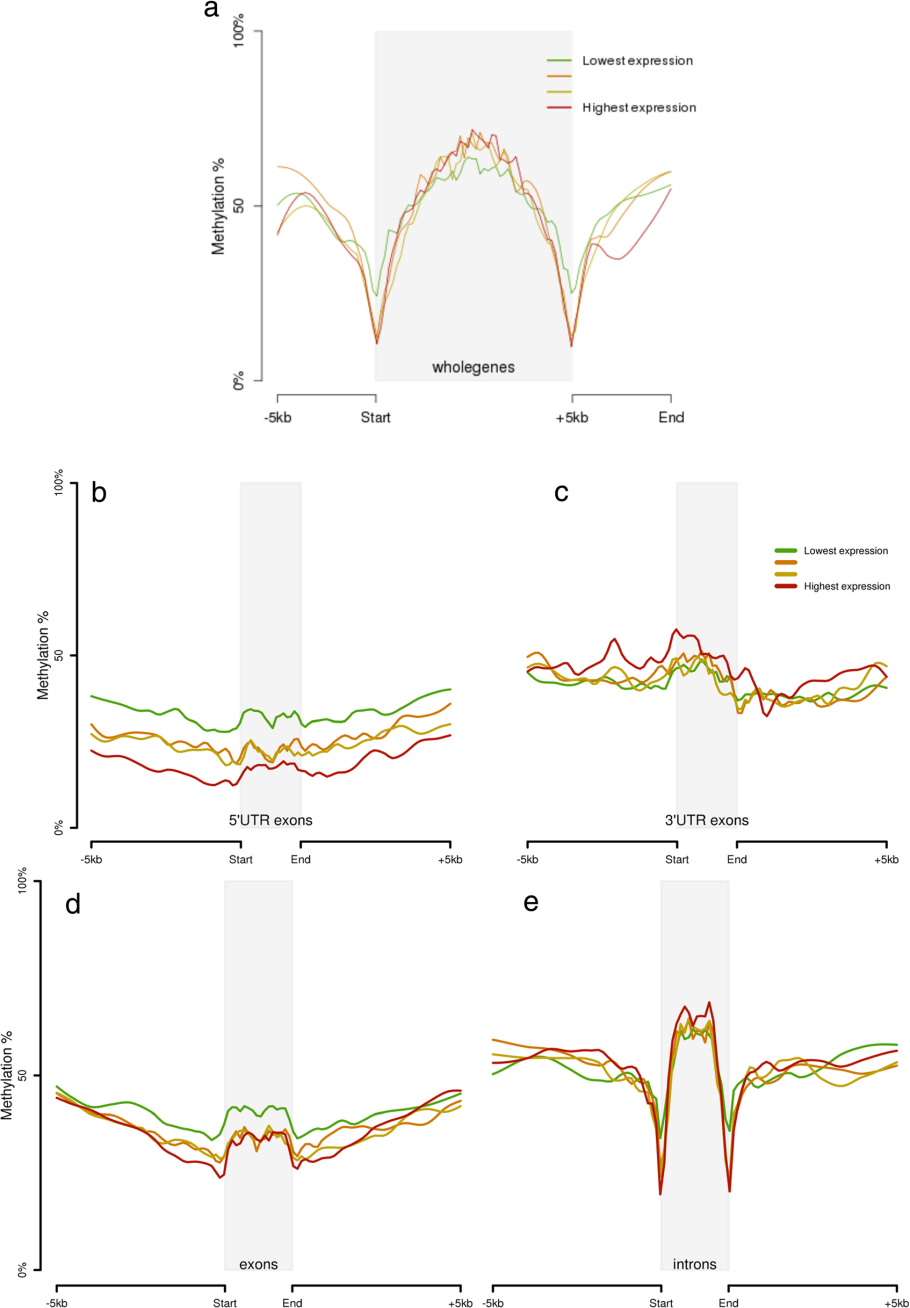
Association between DNA methylation and expression in CLL samples. **a** Local regression showing methylation levels of whole genes stratified by expression quartiles in CLL samples. **b** Local regression showing methylation levels within 5′ and 3′UTRs for different transcripts stratified by expression quartiles. **c** Methylation levels within exons and introns for transcripts in different expression quartiles. **d** Methylation levels at exon boundary in different expression quartiles. **e** Methylation levels at intron boundary in different expression quartiles

Next, we looked at the association between gene expression changes and methylation differences. For this, expression values of all transcripts from 19 matching CLL samples were divided into four expression quartiles and referred to as lowest to highest expression groups. Methylation profiles for genes in each of these quartiles were then extracted for comparison. Figure 5a shows a significant reduction of methylation at gene boundaries for all expression quartile groups. At the peripheral region, methylation in the highest expression genes is reduced the most. Towards the center, methylation of CpGs and expression has an inverse relationship, with the lowest expression genes having the lowest percent methylation. Overall, average methylation for whole gene regions for all genes had a negative correlation (Pearson correlation = −0.07; *p* value = 3.1E-09) with expression.

Next, we looked for expression (transcript FPKM values) and methylation (for overlapped CpG sites) relationship in exons and introns individually (Fig. 5d, e). We observed that among exons (Fig. 5d), transcripts in the lowest expression quartile had the highest and most distinct methylation pattern. Overall, all exons combined from transcripts in all expression quartiles had a negative correlation (corr = −0.13; *p* value <2.2E-16). For introns (Fig. 5e), this relationship appeared to be opposite with the highest expression quartile transcripts showing the highest methylation but almost no correlation (corr = −0.02; *p* value = 6.2E-2). Also, we identified a very clear distinction in methylation patterns from different expression quartiles in exons specifically from 5'UTRs (Fig. 5b). Overall, 5'UTRs had a negative correlation between methylation and expression (corr = −0.2; *p* value <2.2E-16). Conversely, exons from 3'UTRs had the opposite methylation pattern (Fig. 5c). 3'UTR exons from the highest expression quartile genes had the highest methylation (Fig. 5d, Figure S8 in Additional file 1) and overall, they had weak but positive correlation (corr = 0.1; *p* value = 2.4E-3). In summary, we observed different methylation effects on expression for different genic regions, particularly within exons from 5'UTR and 3'UTR that related to methylation changes at widespread genomic locations in CLL.

### Correlation module analysis

In order to further investigate the role of 3'UTR methylation change on expression, we used both expression and methylation data to construct co-expression and co-differential methylation network modules, respectively. These modules were generated using the weighted gene correlation network analysis (WGCNA) framework [40]. For this, we selected 1780 transcripts from 19 matching CLL samples, from which both expression and average 3'UTR methylation data were available.

WGCNA identified 21 co-expression modules with sizes ranging from 41 to 181 transcripts from the expression data and 17 co-differential methylation modules with sizes ranging from 37 to 284 transcripts from the methylation data (average of all CpGs within 3'UTRs). Methylation values of transcripts in each CLL sample were compared against methylation of transcripts from one common control sample, individually. Differential methylation values for all transcripts in all CLL samples were thus obtained. Correlation network modules in each dataset were obtained using hierarchical clustering of pairwise gene correlation structures using WGCNA. WGCNA does not use gene ontology information but clusters the interconnected genes defined as branches of a hierarchical cluster tree. Hence, modules are initially labeled by arbitrary integers and then coded by colors for each dataset. Since clustering for module generation has no gene annotation or functional information, functional interpretation for each module in each dataset was further obtained by conducting a GO enrichment analysis. GO enrichment analyses revealed unique and significant enrichment of various GO terms, providing evidence of a functional role for each module as a whole (Additional file 7; Tables S2, S3, S8, and S9 in Additional file 1). Overall, different biological processes for different modules implied biological significance of clustering transcripts in separate modules in both expression and methylation data.

Next, to investigate the relationship between expression and methylation modules, these modules were matched by pairwise comparison of each methylation module to each expression module using two methods. First, they were compared to measure a statistically significant overlap of genes in each pair. Second, we used network-based statistics to assess whether the density and connectivity patterns of genes were also preserved in a two-paired set of modules with significant gene overlaps. The second method generated a composite statistic value, i.e., *Z*_summary_, using a permutation test to measure the strength of methylation module and expression module preservation. Also, knowing the *Z*_summary_ statistic bias towards a module with a large size, a rank-based statistic *medianRank* was used in the second method to measure their relative preservation irrespective of module size. The medianRank is the statistic calculated from observed preservation values and does not conduct any permutation test against background gene modules.

**Fig. 6:**
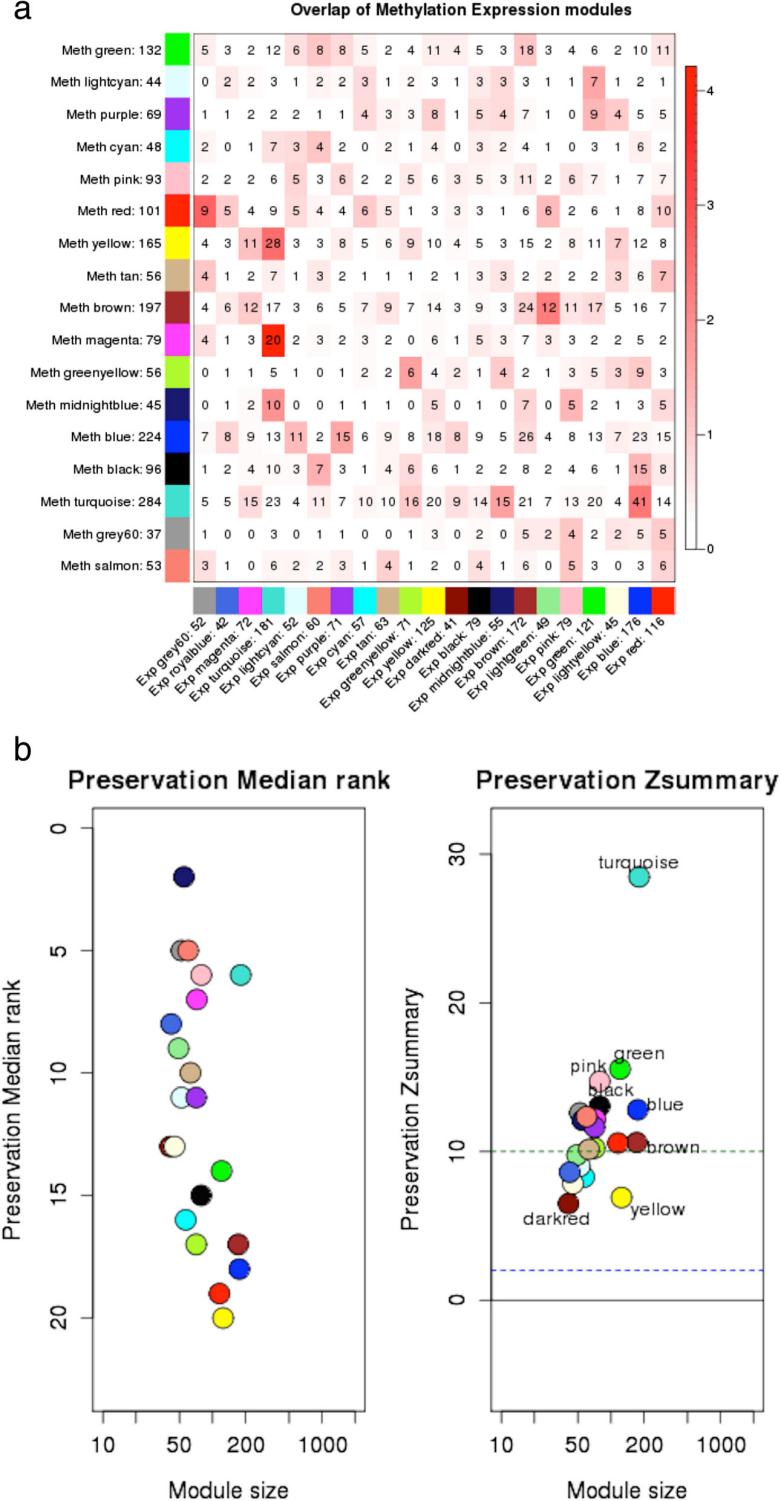
Module preservation. **a** Table showing gene overlap between each pair of methylation and expression modules. Each *row* of the table corresponds to one methylation module (labeled by color as well as text), and each *column* corresponds to one expression module. *Numbers* in the table indicate gene counts in the intersection of the corresponding modules. *Coloring* of the table encodes − log(*p*), with *p* being the Fisher’s exact test *p* value for the overlap of the two modules. The stronger the *red color*, the more significant the overlap is between a methylation module for 3′UTR and an expression module. **b** Plot showing statistical analysis results for module preservation test to check preservation of 3′UTR methylation modules against expression modules based on the density and connectivity patterns of genes in each module. The *left panel* shows the medianRank of the observed preservation statics and the *right panel* shows the distribution of Z_summary_ statistics obtained from a permutation analysis for each methylation module. A module with a lower median rank tends to exhibit stronger observed preservation statistics than a module with a higher median rank. The Z_summary_ statistic of a given module summarizes the evidence that the network connections of the module are more significantly preserved than those of random set of genes of equal size. The significance thresholds for Z_summary_ are Z_summary_ < 2 implies no evidence that the module is preserved, 2 < Z_summary_ < 10 implies weak to moderate evidence, and Z_summary_ > 10 implies strong evidence for module preservation between co-expression module and 3′UTR methylation module

From network preservation tests, we found that expression and differential methylation modules in general exhibited relatively few overlapping genes (Additional file 7) although some of the overlaps were statistically significant. The most significant overlaps (p.adj < 0.05) were observed between large co-expression modules and co-differential methylation modules, enriched for same GO terms (Table 1 and Additional file 7; Table S4 in Additional file 1). Figure 6a reports the number of common genes resulting from pairwise module overlap analysis. The statistical significance of each pair as shown by a color scale was computed to see if the numbers of common genes were obtained according to an iterative pattern and not by chance. WGCNA arbitrarily color-code modules for visual identification, so all color-module associations described here do not have any additional meaning outside of this specific analysis. The most significant overlap as shown in Fig. 6a was for “turquoise” color-coded expression module with “magenta,” “yellow”, and “midnight blue” methylation module. The turquoise expression module also had the highest *Z*_summary_ (*Z*_summary_ = 28) and median rank from network-based statistic method (Fig. 6b). GO enrichment analysis of the turquoise expression module showed enrichment of “*TNFR*,” “cell adhesion,” “leukocyte proliferation,” and “apoptosis/cell death.” All the overlapping methylation modules which were color-coded in magenta (enriched for “*EGF*” p.adj = 1.60E-02), yellow (enriched for “focal adhesion and kinases”), midnight blue (enriched for “cofactor and ion binding”) were enriched for growth factor and regulation of “Ras protein signal transduction,” “kinase activity,” and “ion binding.” Hence, we see that the most significantly overlapping and preserved modules in CLL samples were the ones involved in cancer development. This also indicates the regulatory role of 3′UTR methylation on expression change in cancer.

**Table 1:** Significant overlap and preserved modules in WGCNA of 3′UTR methylation and expression

Methylation module (size)	Expression module (size)	Overlap count (*p* value)	*Z*_summary_	Median rank	Functional annotation	Gene symbols
Magenta (79)	Turquoise (181)	20 (6.17E-05)	28	6	Regulation of Ras protein signal transduction	*ABCC3,ALS2CL,ATG16L2,COG8,DNASE1,DOCK9,ESPL1,PDE4D,TMEM63C,TNFAIP2,TNFRSF4,TTLL1,UQCC1,ZNF385A*
Red (101)	Grey60 (52)	9 (2.04E-03)	13	19	Cell division and chromosome partitioning/cytoskeleton	*CHID1,FDXR,HERC2,HERC6,MYO9B,SEPT5,UBXN7*
Yellow (165)	Turquoise (181)	28 (3.18E-03)	28	6	Kinases	*ATG16L2,BMP1,C1orf159,CLUH,COL6A2,CRTC1,DAGLA,DNAH3,ENTHD2,EPHA10,FAM101B,FAM53A,GPR56,IGF1R,LAMC3,NLRP2,NR3C2,OPLAH,PITPNM2,PPP2R2B,PRKCA,PTPRK,RASGRF1,STK32C,THNSL2*
Turquoise (284)	Blue (176)	41 (4.83E-03)	13	18	Signaling and apoptosis, cell cycle checkpoint	*ACOXL,ANKH,ARHGEF18,B3GAT3,CCAR1,CCDC137,CKB,CREM,DDX39A,DFFB,DNAJB12,FAIM3,HUS1,ITPK1,KIAA0930,LRPAP1,MGRN1,MRPS24,NADK,NRARP,NUBPL,NUP155,OSBPL7,PHF14,PIP5K1A,PPFIA3,PSTPIP1,PTMA,RALBP1,RIN3,RPS6KB2,SH3BP2,SH3TC1,TP53I3,TRAF4,TRIB2,TSSC1,USP42*
Brown (197)	Light green (49)	12 (5.48E-03)	9.8	9	Apoptosis/cell death	*UEVLD,UNC13B,DTNB,BANP,IRF7,CCNL2,INTS9,FNTA,RBM19,DNASE1,GRB7*
Midnightblue (45)	Turquoise (181)	10 (1.24E-02)	28	6	Ion binding	*ABLIM2,ACE,COMMD1,ELFN2,GLT1D1,HAUS7,TOM1*
Turquoise (284)	Midnight blue (55)	15 (2.11E-02)	12	2	Nucleotide binding	*ADCY9,AGTRAP,BSDC1,INO80E,MCHR1,MTHFSD,NADK,P4HB,PDE8A,POR,RAB3A,RPS19BP1,SMUG1,SOX12,STX8*

Also, “green,” “pink,” “blue,” “black,” and “grey60” color-coded expression modules were the other top modules showing a strong preservation (*Z*_summary_ >10) and low median rank (Fig. 6b). All these four modules were again enriched for “zinc ion binding,” “regulation of transcription,” and “apoptosis”. They overlapped with “light cyan,” “midnight blue,” “black,” and “turquoise” methylation modules. Biological processes like “cell division,” “chromosome partitioning/cytoskeleton,” and “GTPase regulator activity” were enriched in both “grey60” (second least rank = 5, *Z*_summary_ = 13) expression module along with its overlapping red methylation module (Additional file 7). An additional network analysis was carried out using expression and average methylation of whole genes (not just 3′UTRs) that are non-coding (i.e., transcripts that do not encode a protein product) along with 3′UTR methylation. Results from this analysis can be accessed in Additional file 7. This non-coding RNA analysis also gave results similar to the 3′UTRs, including overlap of modules enriched for similar cancer-related terms. Hence, we see that significantly preserved expression and methylation modules were enriched for similar cancer-related biological processes like leukocyte proliferation, apoptosis, signal transduction, and cell-cycle regulation. Our observation from network preservation of expression and 3′UTR methylation change provides clues for a better understanding of the contribution made by the regulatory role imposed by 3′UTR methylation overexpression.

**Fig. 7:**
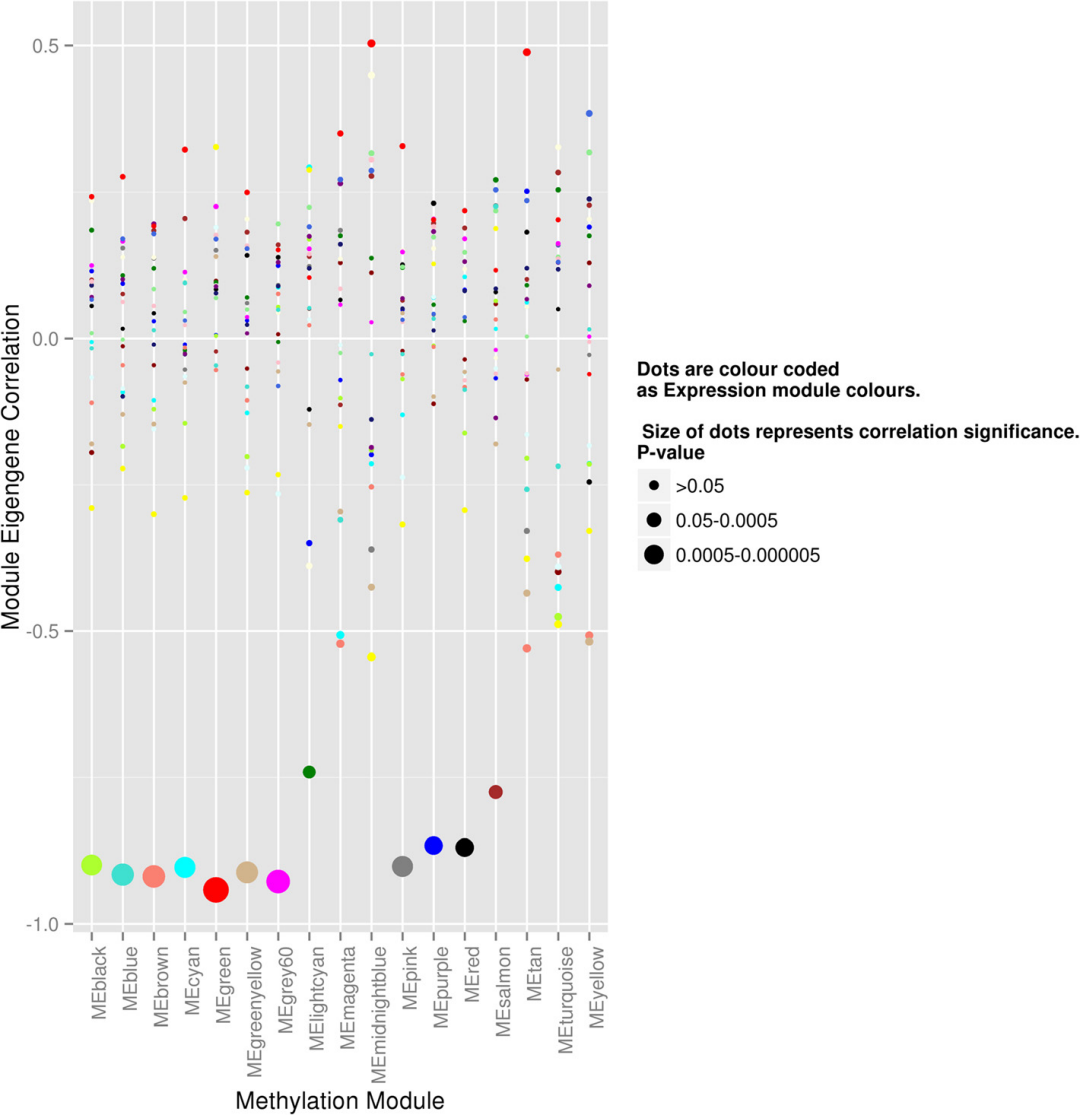
Pairwise correlation between each methylation module to each expression module. Plot showing correlation between eigengenes of each 3′UTR methylation and expression modules. X-axis shows all 17 differential methylation modules, and Y-axis shows the module eigengene correlation value for each of the 21 different color-coded dots representing 21 expression modules. Statistical significance of each correlation was calculated and represented by dot size for each corresponding methylation module

Next, a correlation analysis between methylation and expression modules was conducted using module *eigengene* (aka *eigennode*) that is intuitively understood as a weighted average of the variable profiles in a module. Although the composition of co-expression and co-differential methylation modules can vary, we observed multiple strong Pearson correlations between many expression and methylation module eigengenes as shown in Fig. 7, and Tables S6 and S12 in Additional file 7. For example, in our non-coding gene analysis, eigengenes of “red” methylation module was highly negatively correlated (corr = −0.97, *p* value = 5.75E-12) to a “brown” expression module. The red methylation module was enriched for “regulation of cell cycle” and “intracellular signal cascade” and “brown” expression module for apoptosis and leukocyte proliferation as per GO analysis, showcasing complimentary functional annotations involved in cancer regulation (Tables S8 and S9 in Additional file 7). Similarly, eigengenes of the blue methylation module were significantly and positively correlated (corr = 0.95, *p* value = 1.22E-09) to the “dark red” expression module, both of which were enriched for “protein localization” or “intracellular transport” (Figure S6 in Additional file 7). Also, we saw both significantly positive and negative correlations in 3′UTR methylation to expression modules and occasionally for the same module. For example, red expression module (enriched for “kinases and nucleotide binding”) was negatively correlated with “green” (enriched for “nucleotide binding” and “positive regulation of apoptosis”) and positively correlated with “midnight blue” and “tan” (both enriched for “nucleotide binding”) 3′UTR methylation modules. All together within the 3′UTR methylation correlations, we observed a majority of positive correlated modules of lower significance compared to negative correlated modules of high significance. Correlation in different directions can be assumed to be due to location of methylation differences within exons or introns, as we saw in our previous analysis and the direction of methylation change. Overall, we observed significant correlations in modules enriched for cancer-related terms, giving evidence of the role of methylation change in 3′UTRs towards tumorigenesis.

### Interplay between hypo- and hypermethylation

**Fig. 8:**
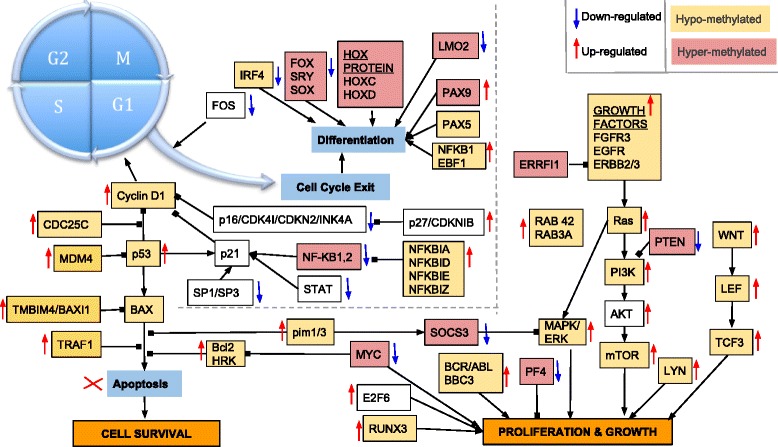
Coordination of hypo- and hypermethylation in cell-cycle regulation in cancer. Plot showing coordination between direction of methylation and expression change in cancer regulation. Each gene is colored showing their methylation change along with *up or down arrow* showing how their expression changes. Genes that are not marked by color or arrow shows no corresponding data recorded

Also, while describing the importance of hypomethylation in CLL, we described the overall interplay among hyper/hypomethylation and gene expression change. Figure 8 shows both methylation and expression change information together for key cancer and cell cycle-regulating genes. Genes are marked as hypo- or hypermethylated if any of the respective C-DMRs overlap on them. As observed from all our enrichment analyses, Fig. 8 shows that growth and proliferation is dominated by hypomethylation whereas hypermethylation blocks cell-cycle exiting and differentiation. We can see many instances where opposite methylation changes target genes in the same process but still coordinate with each other towards cancer development. Both hyper- and hypomethylation changes were found within the same network based on functional role or direction of target gene in the network. For instance, although, all genes involved in cell growth and proliferation are targeted by hypomethylation and have activated transcription, *PF4*—which is known to be involved in inhibition of hematopoiesis and *PTEN*, is hypermethylated. *PF4* negatively regulates the *PI3K-AKT/PKB* signaling pathway and acts as a tumor-suppressor and hence, hypermethylated and repressed. Similarly, in order to drive the cell cycle forward to progress through subsequent phases of cell cycle and escape cell-cycle exit, Fig. 8 shows much complementary coordination of opposite methylation changes. We can see how the *FOS* gene, which is involved in cell-cycle exit is repressed in CLL samples but *CyclinD1* and its other genes, which are required for G1-S phase transition in cell-cycle progression are hypomethylated. Hypomethylation of genes involved in G1-S transition, thereby enables uncontrolled cell division. Also, all genes involved in inhibiting apoptosis are hypomethylated leading to their transcription activation. These examples show how hypo- and hypermethylation coordinate with each other to impose a double negative effect towards the same goal of cancer development in CLL.

## Discussion

### Role of hypomethylation in cell-cycle regulation, histone modification, and transcription activation in CLL

Hypermethylation at the promoter region of tumor suppressors and their subsequent silencing is a well-studied mechanism of tumorigenesis. In contrast, hypomethylation, potentially leading to upregulation of oncogenes, is not fully understood. Also, genic hypomethylation is often considered as a random and non-consistent process due to a particularly predominant de-methylation process in mature B cells in CLL samples. In this study, we showed that consistent hypomethylated regions (referred to here as hypo-C-DMRs) account for a significant pattern of methylation changes in CLL with a distinctive pattern of gene expression and regulatory associations.

In particular, we observed that both hypo- and hyper-C-DMRs were enriched for similar biological processes but in an opposite direction. For example, hyper-C-DMRs were enriched for “positive regulation of cell differentiation”, but hypo-C-DMRs were enriched for “negative regulation of cell differentiation.” We also observed a significant enrichment of *BCR* signaling associated with hypo-C-DMRs. This further strengthens the importance of hypomethylation in CLL since *BCR* is a central pathogenic mechanism in B cell malignancies, including CLL [41]. In addition, GO annotations relate to transcription regulation, chromatin modification, apoptosis, cell proliferation, leukocyte differentiation, and signal transduction were enriched for hypo-C-DMR, which also defines their functional role in cancer development.

Also, the most significantly enriched TFBS for hypo-C-DMRs was *EBF1*, which is a transcription factor that is critical for both B lymphopoiesis and B cell function [42]. *EBF1*, in collaboration with a hierarchy of partner proteins, including E2A, Runx1, and Pax5 (also enriched in motif analysis) activates the B cell transcriptome and represses programs of alternate hematopoietic lineages. DNA binding by *EBF1* has also been linked to changes in epigenetic marks on their target genes. Binding of *EBF1* and other factors including *E2A* have also been correlated with H3K4me1 at target genes, which is also the most enriched histone modification mark in our analysis. H3K4me1 is in fact known to facilitate additional epigenetic modifications necessary for transcription [43]. *RUNX3*, *TCF3*, *PU.1*, and *PAX5* are also key transcription factors in B lymphopoiesis and cell proliferation. Other TFBS enriched for hypo-C-DMRs were *POL2*, *CHD2*, *WHIP*, and *TBLR1. CHD2* is a DNA-binding helicase that specifically binds to the promoter of target genes, leading to chromatin remodeling and promoting their expression [44]. WHIP is a protein that binds to DNA polymerase delta and increases the initiation frequency of DNA polymerase delta-mediated DNA synthesis [45]. *TBLR1* is a key regulator of different properties of the BCL-3 [46] that acts as an oncogenic protein through multiple mechanisms that include the induction of cyclin D1 expression and also inhibits cell apoptosis through induction of the E3 ligase of *p53*, *MDM2* [47, 48]. Among other enriched histone modifications, H3K79 di-methylation is known for regulating the initiation of DNA replication [49], and H3K36me3 is found in actively transcribed gene bodies of genes involved in G1/S transition in a cell cycle [50]. Presence of hypo-C-DMR overlapping enhancers and weak promoters further emphasize their role in activation of transcription. Overall, both enriched TFBS and histone modifications are known to relate to B lymphopoiesis, transcription activation, and cancer development.

In contrast to hypo-C-DMRs, hyper-C-DMRs, which are known to regulate the expression silencing mechanism, were implicated by the enrichment of *EZH2. EZH2* contains a histone methyltransferase SET domain that methylates histone tails on gene promoters to repress their transcription initiation, and this domain is an important component of the polycomb repressive complex 2 (PRC2). The *PRC2* protein *EZH2* is also known to preferentially methylate Lysine 27 on histone 3 (H3K27) [51] and also H3K9 under certain conditions. H3K27me3 and H3K9me3 were both enriched for hyper-LSDMRs in our analysis as well. H3K4me3, H3K9me3, and H3K27me3 co-localizes with most polycomb target proteins like *SUZ12*, *CTBP2*, and *EZH2*-binding sites enriched in hyper-C-DMRs (Additional file 1; Figure S5). Several other studies [51, 52] have reported DNA methylation and tumor suppressors in cancers marked with polycomb proteins enriched with *EZH2* and H3K27me3. This study also elucidates the known mechanism of hyper-C-DMR in gene silencing and promoting cancer development. Further, enrichment of repressor chromatin region for hyper-C-DMRs confirms their role in silencing the expression of target genes.

Our motif enrichment analysis showed hypermethylation enriched for motifs like homeobox and TATAbox, which are usually present in promoter regions and thus silence many key genes. In contrast, hypomethylation was enriched in motifs of transcription activator binding genes, such as *ETS* [38], *ZFX* [37], *cMYC* [39] (Table 1), which are again involved in cell growth, apoptosis, and metabolism, processes necessary for tumor progression. Enriched transcription factor motifs like *Ikaros (IKZF*) and *PU.1* govern B cell lineage priming, which involves changes in histone modifications and chromatin structure of genes encoding molecules important for the establishment of a B cell program [53]. Other significant classes of motifs enriched in hypo-C-DMRs were motifs containing ETS domain in genes like *Elk1* and *Fli* and RHD domain in genes like *NFAT* and *NFkB-p65*. These genes are downstream nuclear targets of *Ras-MAP* kinase signaling and are also known as oncogenic transcription activators, specific to [54] cell survival and proliferation.

### Methylation pattern in exons, introns, and 3′UTRs

In addition to evidence of transcription activation, we observed that hypomethylation in CLL mostly targets intronic, intergenic, and 3′UTR regions. Regarding the relationship between methylation and expression change with respect to genic locations, we found negative correlation for methylation in exons and whole transcript expression within 30 CLL samples. But, this correlation within exons was inconsistent in UTRs. Exons in 5′UTRs seem to act more like promoters, but exons in 3′UTRs had the opposite effect on expression. Hence, these findings suggest that in contrast to a gene expression-inhibiting role of increasing methylation associated with 5′UTR exons, methylation in 3′UTR exons is in fact required in the normal transcription process.

### Regulation of expression by 3′UTR methylation pattern

Our co-expression and co-methylation network analysis revealed that both transcriptome and methylome can be organized into modules. Genes in co-methylation and co-expression modules were found highly enriched for specific gene ontology categories, underscoring their functional importance. Many 3′UTR modules associated with methylation changes were found to have moderate to strong preservation with expression modules. Also, the most preserved module had functional annotations related to signaling and growth and proliferation. Hence, preserved 3′UTR methylation and expression modules revealed the ability of 3′UTR methylation to dictate their expression. The regulatory behavior of methylation change could, therefore, be detected—not only in 5′UTR, promoters, and gene bodies—but also in 3′UTRs in CLL. Also, significantly correlated 3′UTR methylation and expression modules were enriched for biologically important pathways involved in signaling cascade, apoptosis, and cell proliferation. These results provide a fine-grained look at the interaction among 3′UTR co-methylation and co-expression modules altered in CLL.

In summary, we report that hypomethylation of DNA appears to facilitate the aberrant expression of proto-oncogenes/oncogenes, potentially stimulating cell proliferation in CLL. We observed that apart from global hypomethylation of repeat sequences, there also exists site-specific hypomethylation of certain genes and genic regions, especially in genes linked with signaling pathways (e.g., *BCR*, *LYN RAB8A*, *NFKBIB*), chromatin modifications (e.g., *CHD2*, *CHD3*, *SMARCB1*), cell growth and development (e.g., *EBF1*, *EGR1*, *EGFR*, *ERBB2*, *MYC*), apoptosis inhibition (e.g., *BCL2*, *TRAF1*), and promoting cell proliferation (e.g., *CCND1*, *LYN*, *BCL3*). We observed 3′UTRs to possess a high percentage of hypo-DMRs consistent in the majority of our test samples. We report genes with 3′UTR consistent hypomethylation in CLL like *LIF* and *PIM3*. Along with that, we also report genes with consistent hypermethylation in CLL in 3′UTRs like *HMX2* and other genic regions. We also observed that methylation changes at 3′UTR had significant correlation with expression along with overlapping network modules in both datasets. Our findings, thus, suggest that hypomethylation in different genic regions might exhibit a significant deleterious effect on gene expression that results in malignant transformation and/or tumor progression.

## Conclusions

We observed that hypomethylated regions were less consistent over the genome among different samples, in contrast to hypermethylation loci. However, some hypomethylated regions were highly consistent in most of the samples, and their functional analysis revealed their potential biological significance in CLL.

We observed hypomethylation at many genes containing key TFBS involved in cell growth and development, histone remodeling, apoptosis, and cellular proliferation. We found hypomethylation in many key signaling regulators consistent in majority of samples, which do not appear to be random events or a non-specific part of global hypomethylation. In addition, this study contributes to our understanding about the relationship between methylation and expression levels in CLL samples. Results from positional analyses for genic location indicate that the conventional model of methylation regulating expression in an antagonistic manner is most common. However, we also uncovered an interesting and conflicting relationship between methylation and expression for methylation occurring in exons of 3′UTRs. Specifically, we found evidence of a loss of DNA methylation that not only causes genomic instability but also potentially activates many genes mainly in signaling pathways like *BCR* in CLL. Finally, we showed that 3′UTR methylome and transcriptome are organized into biologically meaningful modules with significant correlations and strong-to-moderate preservation of their density and connections between two datasets. The preserved modules were also found as functionally related indicating the role of 3′UTR methylation in expression regulation.

## Methods

### Data sources

Publicly available reduced representation bisulfite sequencing (RRBS) methylation for 30 CLL and three control samples were obtained from the GEO website (http://www.ncbi.nlm.nih.gov/geo/query/acc.cgi?acc=GSE66121). Control samples were CD19+ B cells isolated from peripheral blood of normal controls. Human genome annotation data from the UCSC genome browser (hg19 genome assembly), such as Refseq and UCSC genes, CpG islands, Vista Enhancers, ENCODE Transcription Factor ChIP-Seq, and RepeatMasker Tracks, were used to annotate differentially methylated regions of interest. For genes with multiple isoforms, the longest one was used as the reference. Promoter regions were selected from −2 kb upstream to the transcription start site of each gene.

### Read mapping and % methylation

The bisulfite-treated sequencing reads in DNA methylation data for CD19+ B cells were mapped to bisulfite-converted human genome using Bismark [55] (using Bowtie). Bismark was used also to obtain the genome-wide cytosine methylation calls at a base resolution in the CpG context. Additional file 8 provides read mapping count tables for each sample.

Sequencing reads from RNA-seq experiments were mapped using Bowtie with known ENSEMBL transcripts as gene models. After mapping, FPKM values for each gene/transcript were calculated by Cufflinks [56] and differentially expressed (DE) genes were defined by abs(ln(fold-change)) > 1.5. FDR-corrected *p* < 0.05 for all DE genes were calculated by Cuffdiff [57]. Next, the over enriched GO categories were obtained based on a .05 FDR cutoff using the GO-seq R package.

### DMR calculation

Considering the high correlation between methylation of adjacent CpGs, the methylation information obtained from RRBS data was summarized on 1000 bp tiling windows (step-size 1000 bp) with minimum 3 CpGs and minimum 10 reads mapped on each CpG using the R package, *methylKit* [58]. For DMR calculation, pairwise comparison of 1000 bp tiles in each of the 30 tumor samples against each control normal sample was performed using Fisher’s exact test. From each such test, differential methylation values were obtained only for the regions that were common between CLL and control sample. Thirty such tests were conducted for each control sample. Next, in order to ensure comparable statistics, only those regions that had differential values from each of the 30 tests were used. This gave us 41,421 common regions obtained from the first control sample comparison tests. Similarly, 39,327 and 41,359 regions were obtained from each of the other two control samples.

### Entropy calculation

Also, the methylation entropy across all CLL samples was calculated in order to see probability distribution of methylation changes for each 1000 bp region across all samples. Entropy for each sample was computed as follows:

The methylation vector *m*_*r*_ of region *r* across *N* samples was defined as,

$$ {m}_{r=}{m}_{r,1},{m}_{r,2},\dots, {m}_{r,5},\dots, {m}_{r,N} $$

where *m*_*r,s*_ represents the methylation level in sample *s*. The sum of methylation levels of region *r* in samples (∑_*S* = 1_^*N*^*m*_*r*,*s*_) was treated as a total methylation value. The ratio of methylation level of region in sample relative to the total value was defined as the relative methylation probability, *p*_*s*/*r*_ = *m*_*r*,*s*_/∑_*S* = 1_^*N*^*m*_*r*,*s*_. The original Shannon entropy of the region can be calculated as ${H}_o=-{\displaystyle {\sum}_{S=1}^N{p}_{\frac{s}{r}}{ \log}_2\left({p}_{s/r}\right)}$.

### Enrichment analysis

Enrichment analysis of genes overlapped by C-DMRs for GO categories and KEGG pathways was performed using GOStats R package [59]. Gene set enrichment for gene symbols overlapping hypo-C-DMRs was also performed using GeneDecks [60] to highlight shared descriptors between pairs of genes based on annotations within the GeneCards compendium of human genes.

The epigenomic enrichment analysis of C-DMRs was performed using Genome Runner [61]. Briefly, genomic coordinates of hyper- and hypo-C-DMRs were collected and tested for co-localization with three groups of genome annotation datasets: (1) chromatin state segmentation by HMM from ENCODE/Broad, (2) histone modifications by ChIP-seq from ENCODE/Broad Institute and ENCODE/Stanford/Yale/USC/Harvard, and (3) experimentally validated transcription factor binding sites from ENCODE/Broad Institute and ENCODE/Stanford/Yale/USC/Harvard. Genomic regions annotated by the ENCODE with any functional/regulatory information (~80 % of the whole genome) were used as a “background” to estimate co-localizations that can occur by chance. *p* values were calculated using Chi-square test and corrected for multiple testing using FDR.

Motif analysis was also carried out using Homer [62] software after retrieving sequences around each DMR CpG along with those around non-DMR CpGs randomly chosen as a background.

### Expression in relation to methylation analysis

To associate gene expression changes with methylation differences, all CpG average methylation values were paired with the average expression of transcripts they overlap in all CLL samples. Estimated expression profiles of all transcripts were divided into four expression quartiles and referred to as lowest to highest expression groups within all transcripts. The positional enrichment analysis for each gene region was performed using Homer, and R scripts were used to calculate and plot smoothened density estimates. Locfit library was used for fitting local regression, likelihood models, and related smoothing procedures.

Correlation of whole transcript expression to methylation of CpGs within each specific gene region was calculated by using the Pearson correlation coefficient. For correlation calculation average methylation of CpGs (across all CLL samples) in a specific gene region and average expression of the whole transcript corresponding to these CpGs were used.

### Correlation module analysis

From 19 matching CLL samples from both methylation and expression data, we obtained differential methylation and differential expression (FPKM) for 3′UTRs of 1780 transcripts. For this, whole transcript expression and average methylation for all CpGs in each of its 3′UTRs were used. Differential expression and 3′UTR methylation for each transcript was then computed by comparing each CLL sample individually against one common control sample.

We then used differential expression and methylation value matrices to identify co-expression and co-differential methylation network modules through the WGCNA R package (see Additional file 7 for more details), individually on each dataset. WGCNA computes networks by calculating each gene-to-gene pairwise correlation and interconnection strength by checking number of shared neighbors. It then finally generates modules using hierarchical clustering. Tables S1 and S2 in Additional file 7 provide a list of 10 top hub genes (genes with most significant module membership) in each module, and Additional file 9 consists of module membership values for all genes in each expression and methylation module.

After constructing modules in each dataset, we checked to see if any of the modules in one dataset were preserved in any of the modules from the other data set using two approaches: cross tabulation and network-based statistics. In cross tabulation, overlaps of the constituent genes in each pair of modules from the two data sets were calculated and Fisher’s exact test was used to assign a *p* value to each overlap. In the second method, we used network module preservation statistics (NP) described in and implemented in [63] the WGCNA R package. The NP method not only assesses the significant overlap of genes, but also whether the density and connectivity patterns of modules defined in a reference data set are preserved in a test data set. We considered expression data as reference data and methylation data as test data. This NP statistic test calculates statistic values based on density and connectivity preservation within reference and test modules. From calculated statistic values, the NP test in the WGCNA package module was used to obtain two values, (1) the median rank, which is the rank for the average of the observed preservation static values and (2) a composite module preservation statistic referred to as Z_summary_ using a permutation test. Thus, we reported a *Z*_summary_ for each expression module in the methylation modules.

Also, since WGCNA groups together highly correlated variables to generate modules, we summarized the variable profiles in each module to a single representative, i.e., the module eigengene. The module eigengene, which is defined as the first principal component of the standardized matrix containing variables in the module was used to calculate the correlation between expression and methylation within non-coding genes and 3′UTRs.

Also, since non-coding genes do not translate and do not undergo post-transcription changes (i.e., stripping 3′UTRs) with no defined UTR or a gene body region (or in other words—whole portion can be identified as UTR), we also conducted the same network analysis by including non-coding genes.

### Declarations

This article has been published as part of *Human Genomics* Volume 10 Supplement 2, 2016: From genes to systems genomics: human genomics. The full contents of the supplement are available online at http://humgenomics.biomedcentral.com/articles/supplements/volume-10-supplement-2.

## Additional files

Additional file 1: Additional Figures, Tables and Methods. This is a “docx*”* file, which includes various additional figures, tables and methods that are referred in the main text supporting this research. (DOCX 3304 kb) Additional file 2: List of C-DMRs. This is an “xls” file providing coordinates for hyper- and hypo-C-DMRs common across all control tests in separate excel sheets, respectively. (XLS 117 kb) Additional file 3: Gene lists for C-DMRs. This is an “xls” file providing a list of genes overlapped by common hyper and hypo-C-DMRs in separate excel sheets, respectively. It also consists of excel sheets for genes overlapping different gene regions for hypo- and hyper-C-DMRs, respectively. (XLS 56 kb) Additional file 4: KEGG and GO enrichment analysis results. This is an “xls” format file that provides enrichment test results from GOStats package for KEGG pathways and GO biological processes for both hyper- and hypo-C-DMRs and DMRs from pooled sample analysis in separate excel sheets, respectively. (XLS 916 kb) Additional file 5: Phenotype enrichment analysis results. This is an “xls*”* format file that provides enrichment results for phenotype with different phenotype descriptors, their enrichment *p* values along with their count and names of their corresponding genes sharing that descriptor for both hypo- and hyper-C-DMRs in separate excel sheet, respectively. (XLS 81 kb) Additional file 6: ENCODE enrichment results. This is an “xls” file that provides the ENCODE epi-genomic mark enrichment analysis results from Gm12878 cell line with the name and the description of the datasets, enrichment *p* values that are adjusted for multiple testing using FDR, for hypo/hyper-C-DMRs in separate excel sheets, respectively, along with the name of the regulatory mark. It also consists of enrichment analysis results from all ENCODE cell lines for each regulatory mark in hypo- and hyper-C-DMRs. (XLS 117 kb) Additional file 7: WGCNA. This is a “docx” file that describes correlation modules in details, together with the step-by-step description of WGCNA analysis. WGCNA description includes data cleaning and preprocessing, adjacency topological overlap matrix construction, module detection, calculation of various module measures, and description of preservation statistics methods used. It includes tables and figures for both 3′UTR methylation and non-coding gene data, and their relation with expression of genes analysis. (DOCX 1555 kb) Additional file 8: Read mapping details. This file provides the mapping statistics for all the samples in both RRBS and RNA-seq datasets. Table S1 lists the number of RRBS reads obtained, their % that is uniquely mapped on human genome (hg19) along with total number of CpGs analyzed from each sample. Table S2 lists the number of RNA-seq reads obtained and their % that is uniquely mapped on human genome (hg19) for each sample. (DOCX 124 kb) Additional file 9: Gene module membership. This is an “xls*”* file that provides the matrix for transcript’s module membership or eigengene-based connectivity measure of each transcript for each methylation and expression module for 3′UTRs in separate excel sheet, respectively. (XLS 1395 kb)
