# Supplementary material for: Hypomethylation coordinates antagonistically with hypermethylation in cancer development: a case study of leukemia
Source: Hum Genomics. 2016 Jul 25;10(Suppl 2):18. doi: 10.1186/s40246-016-0071-5 (PMC4965721; doi:10.1186/s40246-016-0071-5)
Supplement: Additional file 1: — Additional Figures, Tables and Methods. This is a “docx” file, which includes various additional figures, tables and methods that are referred in the main text supporting this research. (DOCX 3304 kb) [file 40246_2016_71_MOESM1_ESM.docx]

**Additional Figures and Tables**

**Figure S1: Histogram plots showing percentage of tiles differentially methylated in each CLL sample on X-axis when compared against different control sample (SC1, SC2 and SC4_1) on Y-axis.**


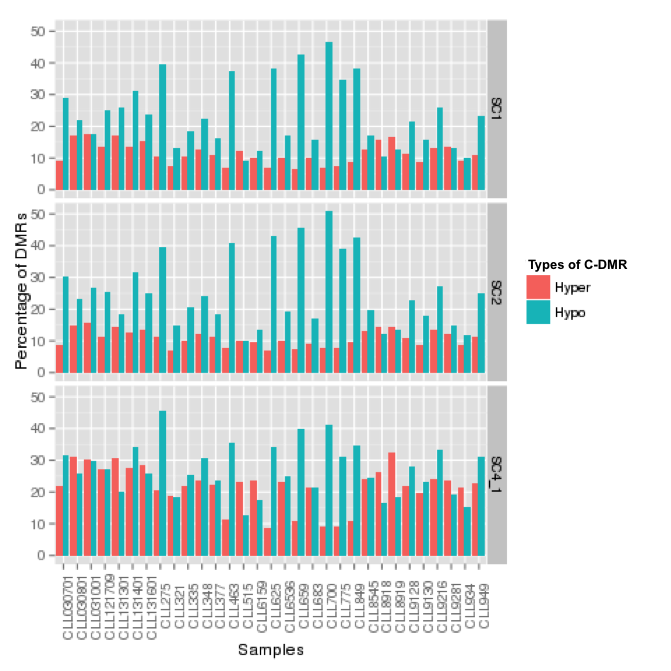


**Figure S2: Chromosomal distribution of common C-DMRs. (a) Percentage distribution. (b) DMR location distribution.**


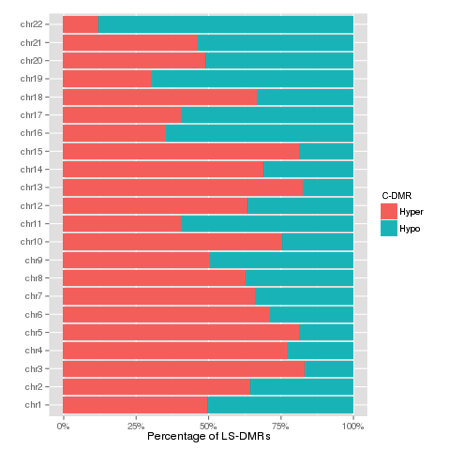

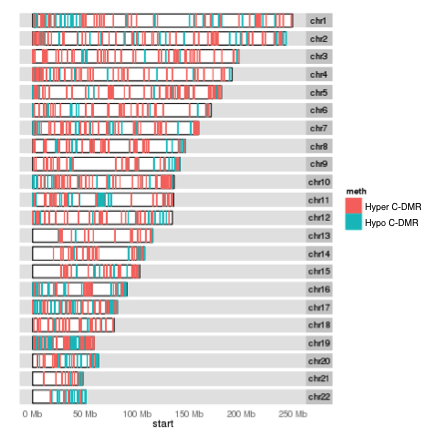


**Figure S3: Enrichment analyses. The ENCODE genome annotation datasets for all cell lines from Broad and Stanford/Yale/USC/Harvard were used for transcription factor and Histone enrichment analyses (a, b), Broad datasets were used for chromatin states enrichment (c). Y-axis shows –log10-transformed enrichment p-values, FDR corrected. Left/right parts of the barplots show top 10 most significant enrichments for hyper/hypo C-DMRs, respectively.**

(a) Transcription Factor Binding Sites enrichment

**
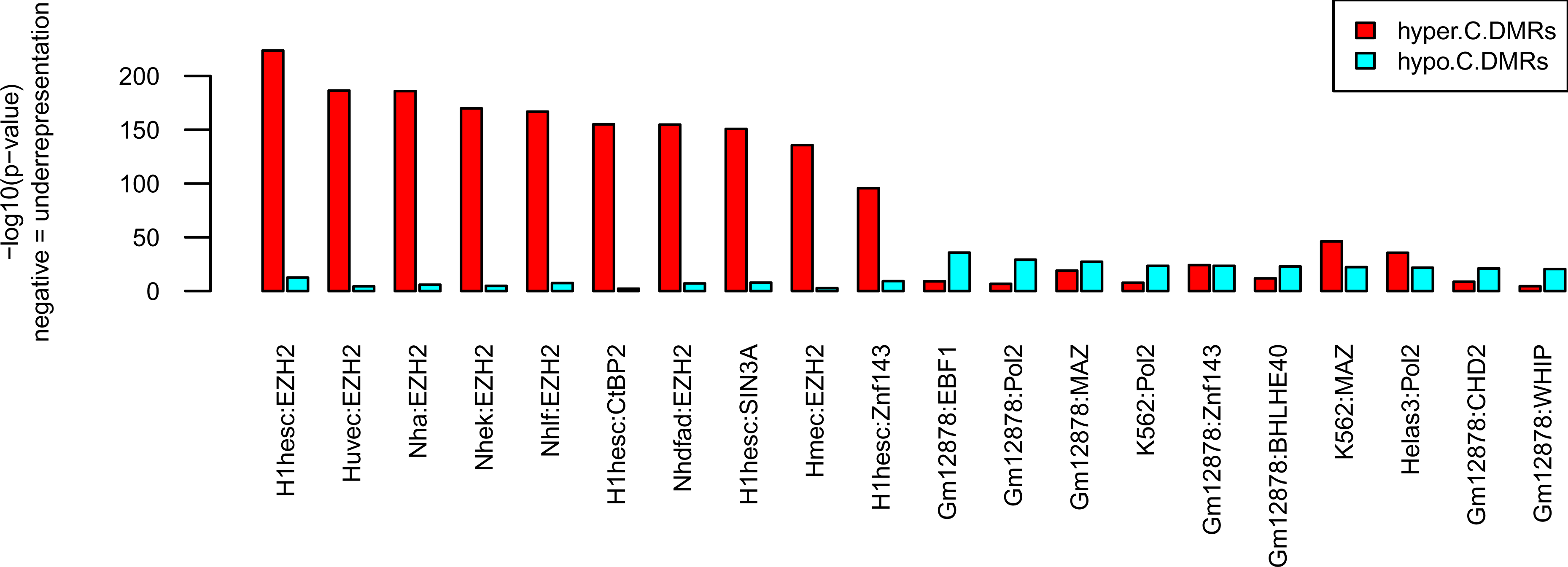
**

(b) Histone modification sites enrichment

**
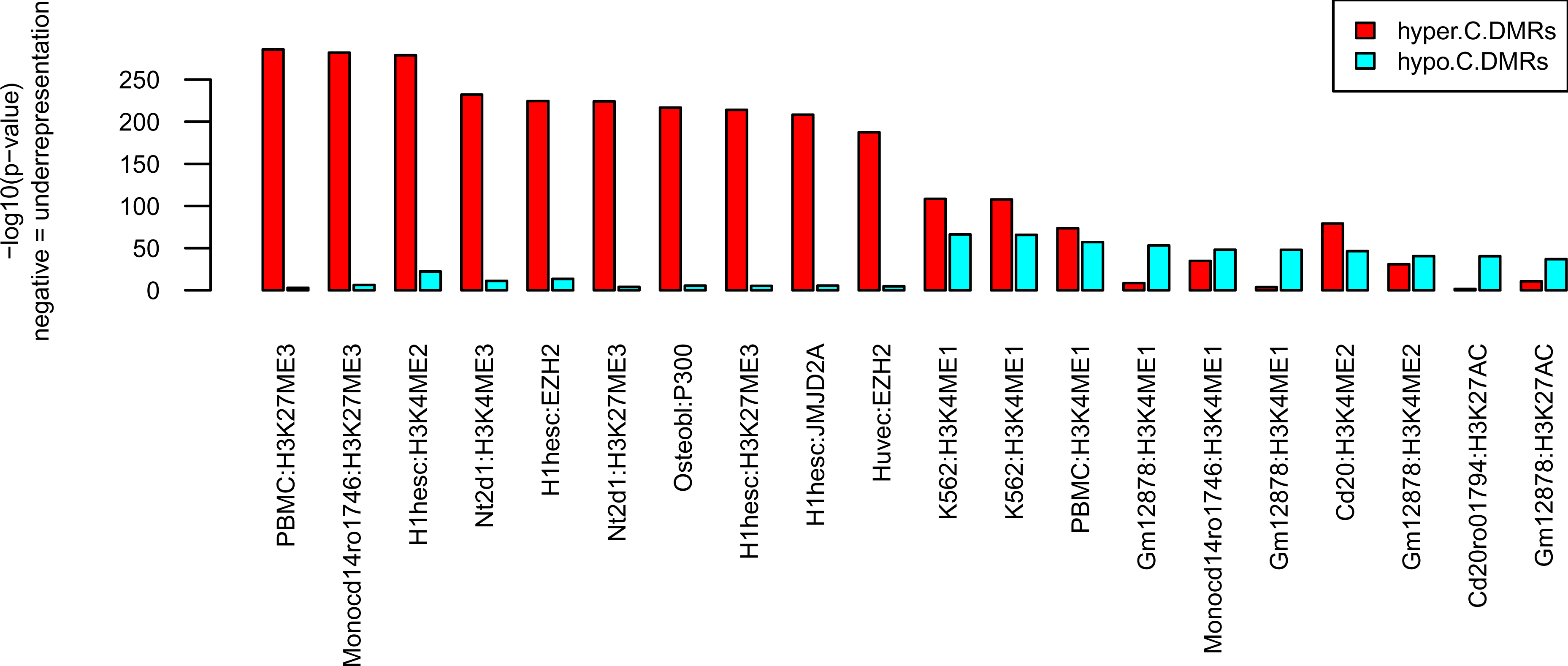
**

(c) Chromatin States enrichment

**
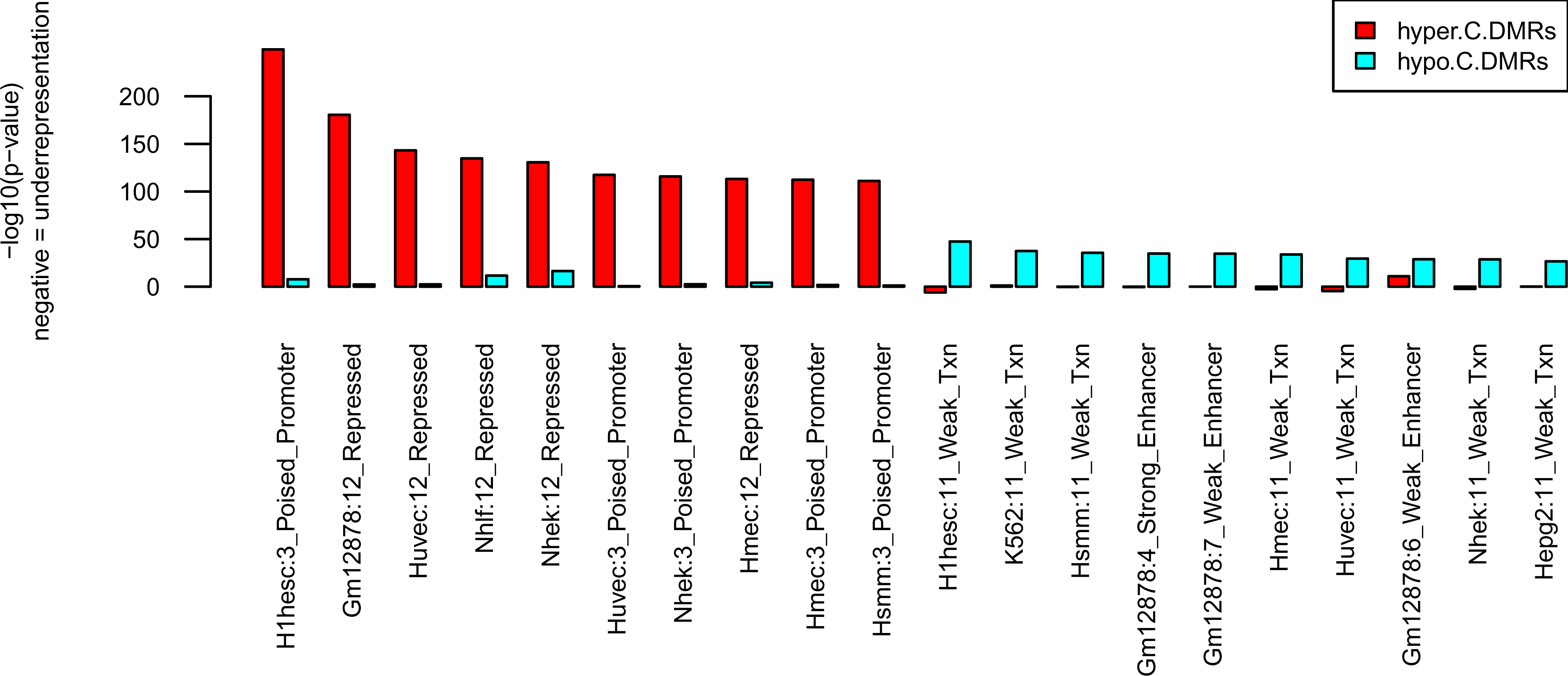
**

**Figure S4: Fold enrichment (log10 converted) of 161 TFBS in CLL sample against hyper and hypo C-DMRs.** ENCODE peak file, wgEncodeRegTfbsClusteredV2 having combined data from different cell lines was used for enrichment. SUZ12, CTBP2 and EZH2 can be seen highly enriched for hyper C-DMRs and EBF1, BATF, BCL3, BCL11A, BCLAF1, IRF4, IKZF1, MEF2A, MEF2C, MTA3, NFATC1, POU2F2, RUNX3, STAT5A, TBL1XR1, TCF3, and WRNIP1.


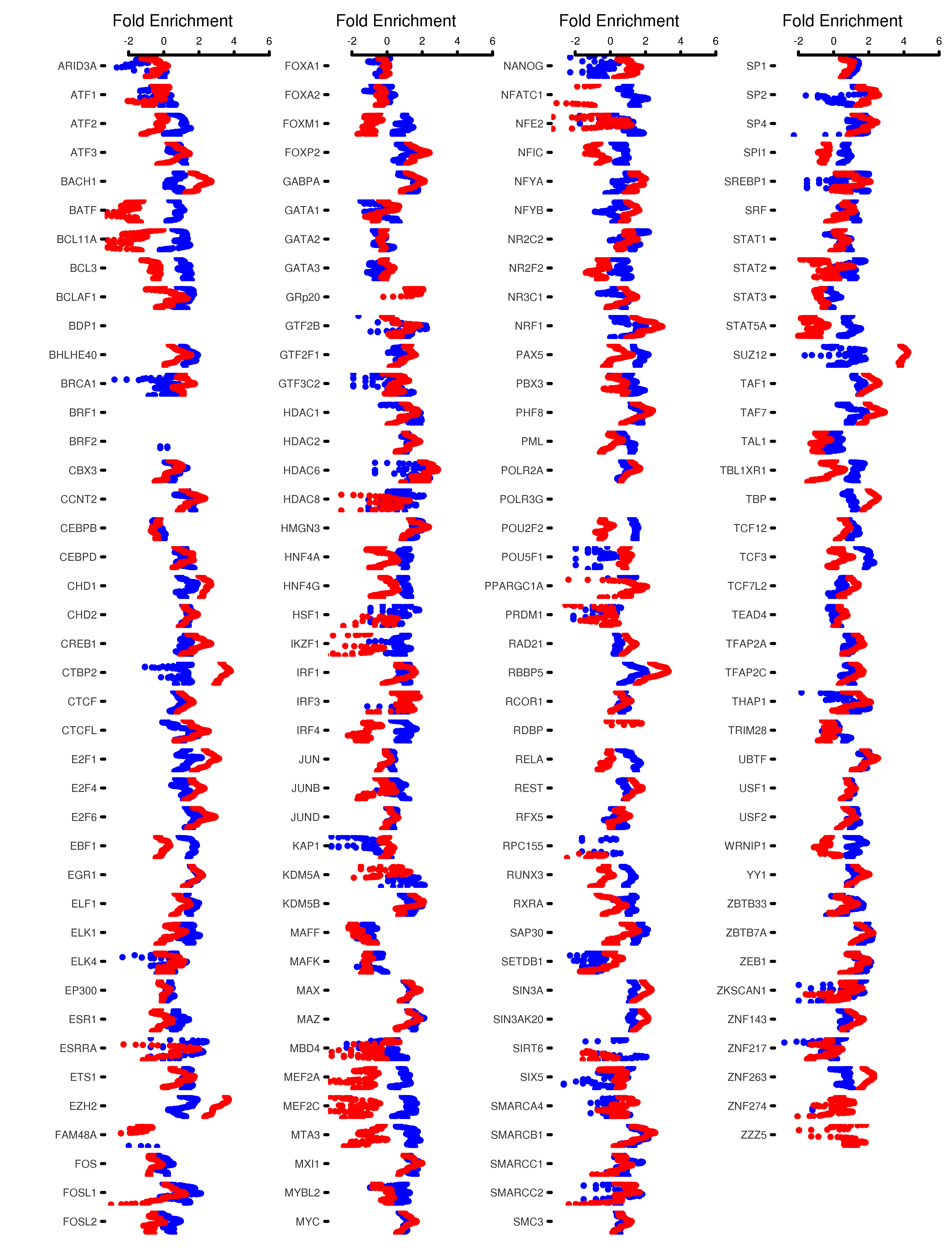


**Figure S5:** **Positional enrichment of enriched ENCODE TF-binding sites** **with chromatin modification marks.** (a,b,c) Positional analysis showing enrichment of H3K4me3 at SUZ12, CTBP2 and EZH2 binding sites, respectively.


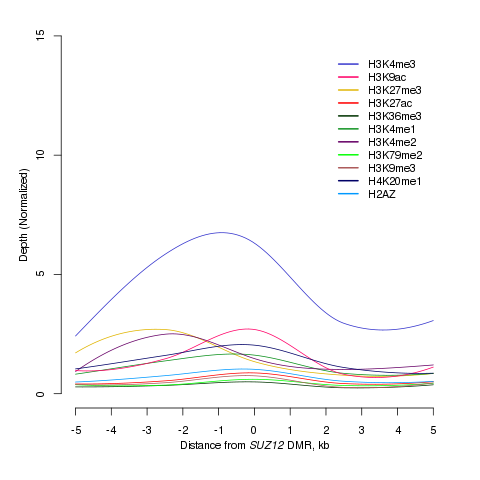

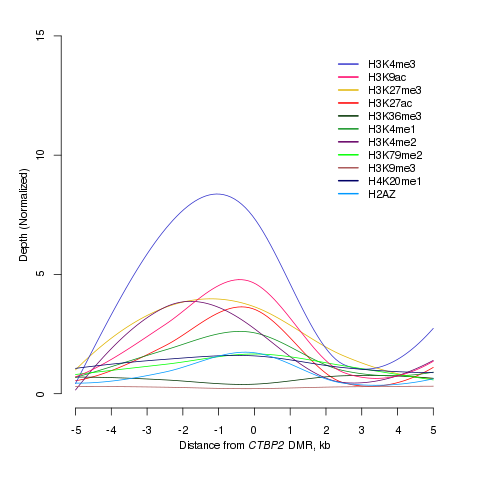

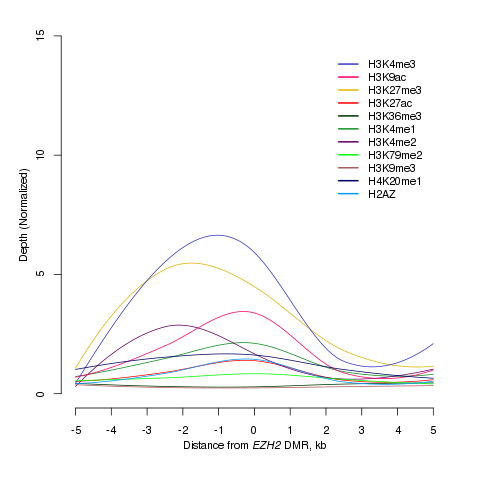


**Figure S6: Distribution of C-DMR CpGs overlapping enhancers over different genic regions.**

**Figure S7: Volcano plot for expression data**


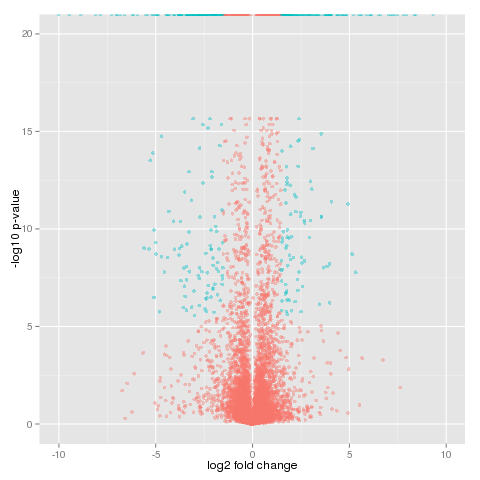


**Figure S8: Binned average expression and methylation scatted plot.** Average CpG methylation (over 10% bin size) on X-axis is plotted against their overlapped transcript average expression on Y-axis for CpGs mapped on different genic regions (marked by different colors). Number of CpGs within each methylation bin is shown by scatter plot marker size. Scatter plot smoothening and curve fitting were done using LOESS regression (non-linear) method. Red line for 3’UTR exons shows very different pattern compared to others with highest expression for CpGs with high methylation.

**
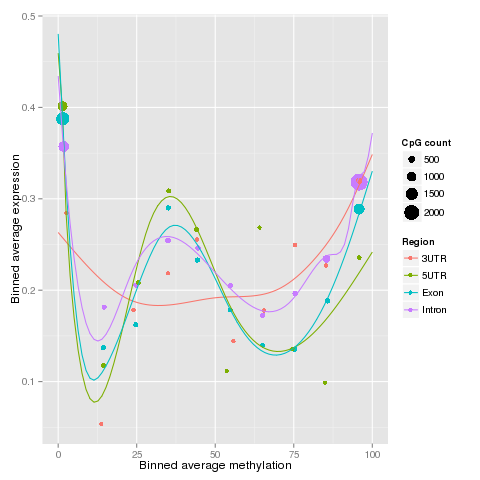
**

**Table S1: Number of C-DMRs that passed binomial test with q-val<0.01.**

|  | All common tiles | Hyper C-DMRs | Hypo C-DMRs |
| --- | --- | --- | --- |
| SC1 | 41421 | 1684 | 1522 |
| SC2 | 39327 | 2079 | 2338 |
| SC4_1 | 41359 | 11749 | 3062 |

**Table S2: Number of DMRs in group-comparison of all CLL samples against different Control sample combinations.**

| **Hypo** | **Hyper** | **Control** | | | **Case** |
| --- | --- | --- | --- | --- | --- |
| 16,190 | 8,056 | SC1 | SC2 |  | All 30 samples |
| 12,786 | 7,120 | SC1 |  |  | All 30 samples |
| 13,150 | 6,662 |  | SC2 |  | All 30 samples |
| 14,965 | 20,637 |  |  | SC4_1 | All 30 samples |
| 15,599 | 17,811 | SC1 | SC2 | SC4_1 | All 30 samples |

**Table S3: Number of conserved DMRs from comparison of each CLL sample against each Control sample.**

| Tests | Hyper in >=25 | Hypo in >=25 |
| --- | --- | --- |
| Comparison against SC1 | 1411 | 1265 |
| Comparison against SC2 | 1796 | 1973 |
| Comparison against SC4_1 | 10485 | 2576 |

**Table S4: C-DMR intersection from tests using different control samples.**

| Common tiles | Hypo C-DMRs | Hyper C-DMRs |
| --- | --- | --- |
| SC1, SC2 | 790 | 1050 |
| SC1, SC4_1 | 853 | 1197 |
| SC2, SC4_1 | 1147 | 1518 |
| SC1, SC2, SC4_1 | 658 | 982 |

**Table S5: Motif enrichment.**

| **Name** | | **Motif** | **P-value** |
| --- | --- | --- | --- |
| **Hyper LS-DMRs** | | | |
| Homeobox (*Lhx2, Lhx3, Nkx6, HOXD13, HOXA9, HOXB4, HOXC9, HNF6, Cdx2, NANOG, lsl1*) | 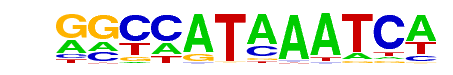 | | 1e-7 |
| E2F*(E2F4,E2F1,E2F)* | 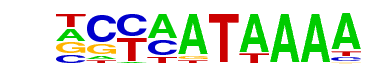 | | 1e-5 |
| CHR/Cell-Cycle-Exp | 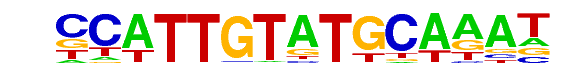 | | 1e-2 |
| TATA-Box/Promoter | 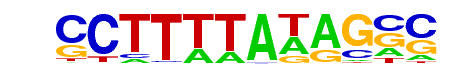 | | 1e-1 |
| **Hypo LS-DMRs** | | | |
| ETS (*Ets1,Elk1, Elk4, Elf1, Elf5, Fl1, GABPA*) | 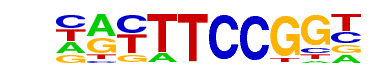 | | 1e-8 |
| RHD (*NFAT, NFkB-p65, NFkB-p50, NFkB-p65-Rel*) | 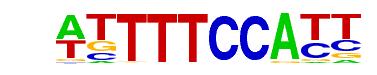 | | 1e-7 |
| POU/Homeobox (*Oct2, Oct4*) | 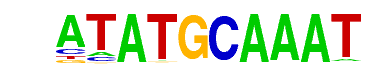 | | 1e-5 |
| Runt (*RUNX1, RUNX-AML, RUNX*) | 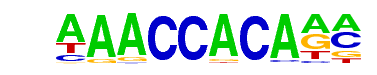 | | 1e-5 |
| IRF (*IRF4, IRF2, PU.1-IRF*) | 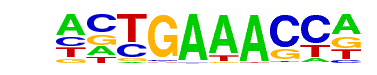 | | 1e-4 |
| HLH (*Tcf12, BMAL1, n-Myc, Ahr, Ptf1a, E2A, MyoG, Ap4, Myf5, E2A-NEAR PU.1, BHLHE40, SCL, MAX*) | 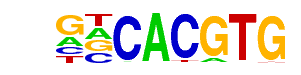 | | 1e-3 |
| Zf (Egr1, Egr2, ZFX, PRDM1, ZNF711, CTCF, GLI3, BORIS) | 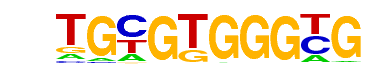 | | 1e-3 |
| PAX5-shortForm (Paired/Homeobox) | 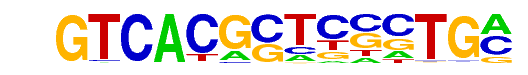 | | 1e-3 |
| bZIP (BATF, ATF3, Jun-AP-1, AP-1, MAFA) | 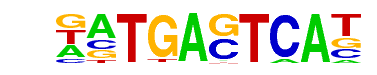 | | 1e-3 |
| NR (Erra, AR-halfsite(NR)/, Esrrb) | 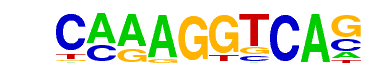 | | 1e-2 |

**Methods**

**Permutation tests**

**Sample Permutations** In our first permutation test, we see if a pattern of more than half of samples to be consistently hypo/hyper methylated for a region is significant or can be found just by chance. In each permutation, samples of both cancer and normal patients were shuffled together and divided into 31 samples as Cancer and 4 samples as normal. From each these two sets, one sample is picked randomly to perform differential methylation test on each of their regions and then calculate the proportion of differentially methylated samples out of 31 rounds.  In other words, for each region i, we count the percentage of hyper/hypo methylated samples, phat_i^1, and record it and then do another permutation, we get phat_i^2. After 1000 permutations, and we get phat_i^1, phat_i^2, …, phat_i^1000 for each region i, which gives the null distribution of the phat_i. Next, we calculate the number of permutations, for which regions with percentage of samples that are hypomethylated in permuted data is more than in un-permuted data out of 1000 permutations, and used to calculate p-value for each region.

P-value of region, i: p-value_i=#{P_i_ > P_i,obs_}/1000

P-value thus obtained tells if the pattern obtained from our CLL data is significant or not. This permutation method shows whether the observed pattern is significant or just by chance.

**Row permutations** In our second permutation test, we want to show the significance of C-DMRs compared to other randomly methylated regions. Here, we consider all regions are independent and there is no pattern for any particular region, and therefore shuffle methylation among all regions in each sample. Shuffling the methylation values in each row in matrix A (but containing raw methylation value), in other words is exchange of labels on observed data points. By doing that we change the methylation values for each region preserving the distribution of methylation within the sample (row) but break any association between methylation of regions across samples. After shuffling regions in all samples, we perform differential methylation test between each of the CLL sample and normal sample for each region. We calculate differential methylation for 100 times and count the number of times percentage of samples hypomethylated is greater than in unshuffled original data for that region. In other words, if P_i,obs_ is the percentage of samples hypomethylated in original data and if for j^th^ permutation, P_i,j_ is the percentage of samples hypomethylated, then P-value for region, i = #{P_i_ > P_i,obs_}/100.

**KEGG and GO Enrichment analysis of pooled samples**

KEGG enrichment analysis of pooled sample DMRs also showed a strong enrichment of signaling pathways like “Calcium signaling pathway” (p.adj=2.53E-03), “MAPK signaling pathway“ (p.adj=1.30E-02), “mTOR signaling pathway” (p.adj=2.89E-02), “VEGF signaling pathway” (p.adj=3.82E-02), etc., and cancer-related terms like “Axon guidance” (p.adj=2.54E-3), “Pathways in cancer” (p.adj=1.30E-2), etc. Hyper DMRs were enriched for terms like “Pathways in cancer” (p.adj=7.95E-06) and “Wnt signaling pathway” (p.adj=3.42E-3).

From GO Biological processes enrichment analysis, similar but more general terms like “neurogenesis” (p.adj=1.31E-10), “regulation of cell communication” (p.adj-5.76E-07), “cell differentiation” (p.adj=1.66E-06), “regulation of signal transduction” (9.22E-06) and “histone methylation” (p.adj=2.42E-02) were found for hypo DMRs. For hyper DMRS, terms like “cellular metabolic process” (p.adj=9.25E-19), “cellular component organization” (p.adj=1.30E-12), “gene expression” (padj=1.67E-11), “regulation of cell communication” (p.adj=1.48E-06), and “regulation of signaling” (p.adj=2.51E-06) were found.

From GO Molecular functions enrichment analysis, “cytoskeletal protein binding” (p.adj=4.40E-08), “kinase activity” (p.adj=4.34E-06), and “histone methyltransferase activity” (p.adj=1.09E-03) were enriched. For hyper DMRs, similar terms like “sequence-specific DNA binding” (p.adj=3.55E-07) were enriched.
