# Supplementary material for: Hypomethylation coordinates antagonistically with hypermethylation in cancer development: a case study of leukemia
Source: Hum Genomics. 2016 Jul 25;10(Suppl 2):18. doi: 10.1186/s40246-016-0071-5 (PMC4965721; doi:10.1186/s40246-016-0071-5)
Supplement: Additional file 7: — WGCNA. This is a “docx” file that describes correlation modules in details, together with the step-by-step description of WGCNA analysis. WGCNA description includes data cleaning and preprocessing, adjacency topological overlap matrix construction, module detection, calculation of various module measures, and description of preservation statistics methods used. It includes tables and figures for both 3′UTR methylation and non-coding gene data, and their relation with expression of genes analysis. (DOCX 1555 kb) [file 40246_2016_71_MOESM7_ESM.docx]

**WGCNA analysis: Correlation module construction**

In order to construct networks, R package for WGCNA was used. Weighted networks have the advantage of preserving the continuous nature of co-expression and co-methylation information, which is particularly useful when studying module preservation. Following the step by step procedure:

1. **Data Cleaning and preprocessing**

- Read datasets in matrix structure with corresponding gene names and sample names.
- Check that the data has the correct format for many functions operating on multiple sets:
- Check that all genes and samples have sufficiently low numbers of missing values.
- Removed the offending genes.

–1780 genes had both methylation in 3’UTR(6302 including non-coding transcripts and) and expression data.

- Generate matrices for methylation and expression with a concordant genes
- Choose a set of soft-thresholding powers
- Constructing a weighted gene network entails the choice of the soft thresholding power β to which co-expression similarity is raised to calculate adjacency. The authors of R package have proposed to choose the soft thresholding power based on the criterion of approximate scale-free topology

Hence, 6 was used as thresholding power to generate adjacency matrix.

1. **Construct Adjacency Matrices**

First the adjacency matrices were constructed, encoding whether/how a pair of node is connected in both methylation and expression dataset. Here, the nodes were genes whose both methylation and expression values were measured in our dataset. For a weighted network, the adjacency matrix reports the connection strength between gene pairs in contrast to un-weighted network where entries are 1 or 0 depending on whether or not 2 nodes are adjacent (connected). For the adjacency or co-expression network, concordance of gene expression was measured with a Pearson correlation and then transformed continuously with the power adjacency function for weighted networks. Power adjacency function for constructing unsigned and signed weighted gene co-expression networks are:

Default values: beta=6 for unsigned and beta=12 for signed networks.

1. **Topological overlap matrix (TOM) and corresponding dissimilarity**

Pairwise measure of inter-connectedness within each module is the topological overlap measure consisting of direct connection and shared neighbours.


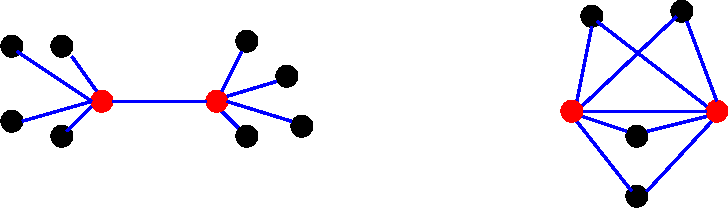


No shared neighbours: low TOM many shared neighbours: high TOM

After adjacency matrices, Topological overlap matrix (TOM) and corresponding dissimilarity were obtained using following formula:

Scaling of Topological Overlap Matrices to make them comparable across sets

Topological Overlap Matrices of diﬀerent data sets may have diﬀerent statistical properties. Hence, scaling of Topological Overlap Matrices is done to make them comparable across sets. Methylation TOM is scaled such that the 95^th^ percentile equals the 95^th^ percentile of the female TOM.

Next, the dissimilarity matix is generated:

where, k=Gene connectivity=row sum of adjacencies

- - For unweighted networks=number of direct neighbors
  - For weighted networks= sum of connection strengths to other nodes

We can see various small clear clusters forming in TOM matrix for Expression but one big turquoise clustering structure in methylation TOM matrix.

1. **Module Detection**

From TOM matrices, groups (clusters) of densely interconnected genes are obtained which are referred as Modules. Modules are the often-defined using average linkage hierarchical clustering that inputs a measure of interconnectedness often the topological overlap measure. Once a dendrogram is obtained from a hierarchical clustering method, modules are defined as branches using a branch cutting method.

Next, the modules are then either labeled by integers (1,2,3…) and equivalently by colors (turquoise, blue, brown, etc.) and all module genes are assigned the same color.

1. **Calculate various module measure**

For each module, **Intramodular connectivity**, *ie.* *kIN* is calculated as row sum across genes inside a given module as follows:

, that can be defined for any network based on adjacency matrix but strong depends on module size.

**Module eigengene** is then defined as the most highly connected gene in a module. **Module membership measure**, also known as *kME* or Eigengene-based connectivity is calculated by :

*kME(i)* is simply the correlation between the i-th gene expression profile and the module eigengene. *kME* close to 1 means that the gene is a hub gene. This can be used as a very useful measure for annotating genes with regard to modules.

R function blockwiseModules (in WGCNA library) using multithreading was used, that implement 3 steps:

1. Variant of k-means to cluster variables into blocks
2. Hierarchical clustering and branch cutting in each block
3. Merge modules across blocks (based on correlations between module eigengenes)

This method was used for module detection in very large data sets. It worked for thousands of variables particularly constructing networks for transcripts for which methylation was covered anywhere in transcripts. But in cases, transcripts with specific region methylation data in 3’UTRs or 5’UTRs, step by step construction could also be used.

1. **Module preservation**

There are two approaches to determine network/module preservation: cross-tabulation and network based statistics.

1. **Cross-tabulation method**: Overlaps of each pair of consensus modules are calculated and used the Fisher’s exact test (also known as hypergeometric test) to assign a p-value to each of the pairwise overlaps. To display the p-value and count tables in an informative way, a color-coded table of the intersection counts is created where colors indicate the p-value signiﬁcance.
2. **Network Preservation Statics based**

The other method to get the preservation of connectivity patterns between nodes is by using Network based statistics. Network based statistics do not require the module assignment in the test network but require the user to input network adjacency matrices.

We distinguish the following 3 types of network based module preservation statistics, they are:

1. **Density based** preservation statistics (*meanCor, meanAdj, propVarExpl, meanKME*) used to determine whether reference module nodes remain highly connected in the test network.
2. **Separability based** statistics (*separability.ave*, *separability.ME*) used to determine whether reference network modules remain distinct (separated) from one another in the test network.
3. **Connectivity based** preservation statistics (*meanCor*, *meanAdj*, *propVarExpl*) used to determine whether the connectivity pattern between nodes in the reference network is similar to that in the test network.

A significance level (p-value) is then generated for preservation statistics, by using a permutation test procedure, which randomly permutes the module assignment in the test data. Based on the permutation test, the mean and variance of the preservation statistic are also estimated, under the null hypothesis of no relationship between the module assignments in reference and test data. And finally, the
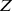
statistic for each preservation statistic is also defined by standardizing each observed preservation with regard to the mean and variance. Under certain assumptions, each 
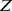
 statistic (approximately) follows the standard normal distribution if the module is not preserved. Hence, the higher the value of a Z statistic, the stronger the evidence that the observed value of the preservation statistic is significantly higher than expected by chance.

Since, preservation statistics measure different aspects of module preservation, different module preservation statistics are aggregated into composite preservation statistics. We define several composite statistics.

For correlation networks based on quantitative variables, the 4 density-preservation statistics are summarized by 
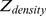
, the 3 connectivity-based statistics are summarized by 
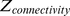
, and all individual 
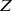
 statistics are summarized by *Z_summary_* defined as follows:


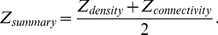


Since biologists are often more familiar with p-values as opposed to Z statistics, R implementation in function modulePreservation also calculates empirical p-values. Analogous to the case of the Z statistics, the p-values of individual preservation statistic are summarized into a descriptive measure called 
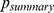
. The smaller 
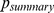
, the stronger the evidence that the module is preserved. In practice, there is an almost perfect inverse relationship (Spearman correlation 
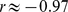
) between 
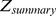
 and 
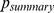
.

The Z statistics and permutation test p-values often depend on the module size (i.e. the number of nodes in a module). This fact reflects the intuition that it is more significant to observe that the connectivity patterns among hundreds of nodes are preserved than to observe the same among nodes of small modules. But in cases when preservation statistics of modules of different sizes are to be compared, the observed values of the individual statistics should are used or alternatively by summarizing them using the composite module preservation statistic median Rank.


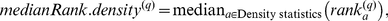


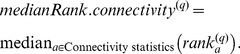


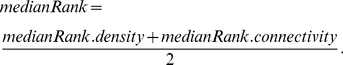


 The Median rankis useful for comparing relative preservation among multiple modules: a module with lower median rank tends to exhibit stronger observed preservation statistics than a module with a higher median rank. Since median rank is based on the observed preservation statistics (as opposed to Z statistics or p-values), it is much less dependent on module size.

**Figure S1: Summary network indices (y-axes) as functions of the soft thresholding power (x-axes). Numbers in the plots indicate the corresponding soft thresholding powers. The plots indicate that approximate scale-free topology is attained around the soft-thresholding power of 6 for both sets. Because the summary connectivity measures decline steeply with increasing soft-thresholding power, it is advantageous to choose the lowest power that satisﬁes the approximate scale-free topology criterion.**

(a) Non-coding genes


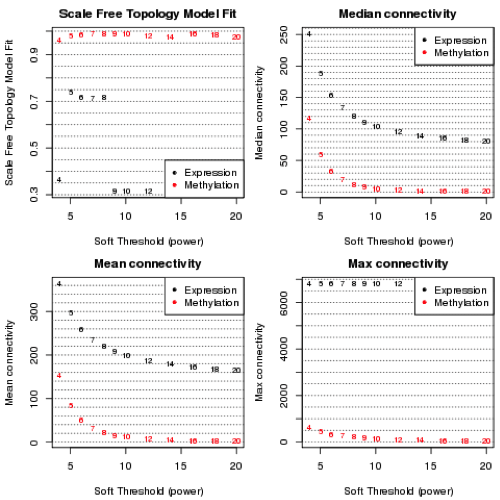


(b) 3’UTRs


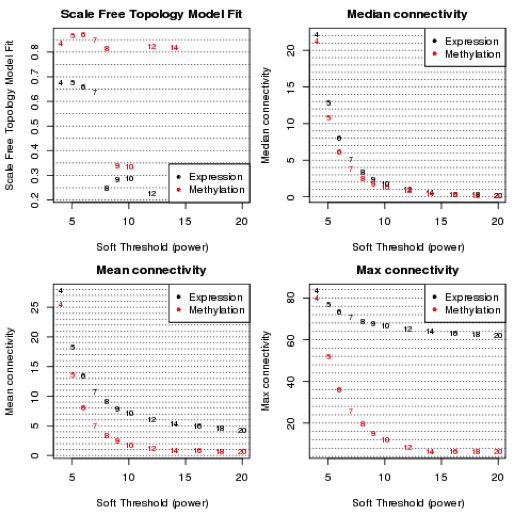


**Figure S2: TOM Heatmap plots (a) For expression and methylation data of all transcripts in independent datasets. (b) For expression and methylation data of consensus transcripts that have 3’UTR methylation data (including non-coding genes). (c) For expression and methylation data of consensus transcripts that have 3’UTR methylation data.**

**
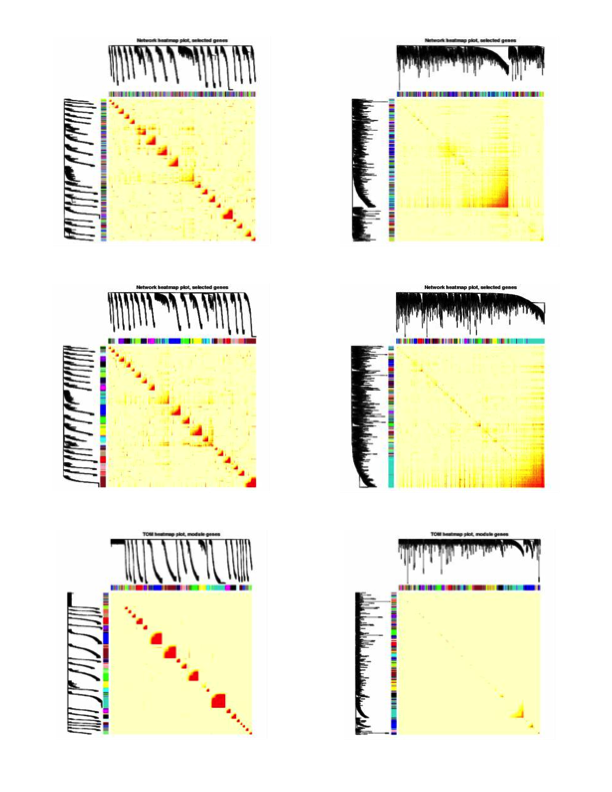
**

**Figure S3: Clustering dendrogram transcripts for independent data sets with their corresponding modules and module colors and dissimilarity based on topological overlap. (a) Expression and methylation dendrogram for all transcripts. (b) Expression** **dendrogram transcripts with 3’UTR methylation data (including non-coding transcripts). Methylation dendrogram for non-coding transcripts and 3’UTRs. (c) Expression** **dendrogram for transcripts with 3’UTR methylation data and methylation dendrogram for 3’UTR methylation.**

(a)
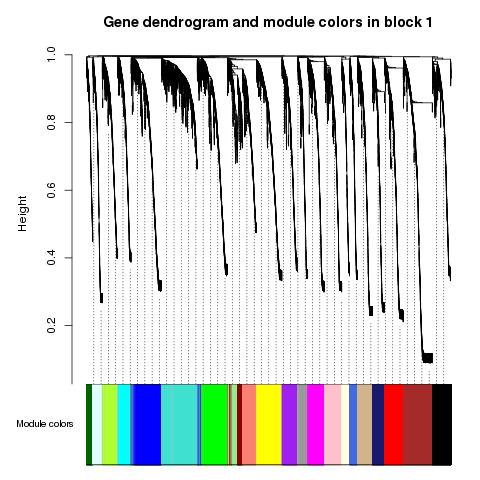

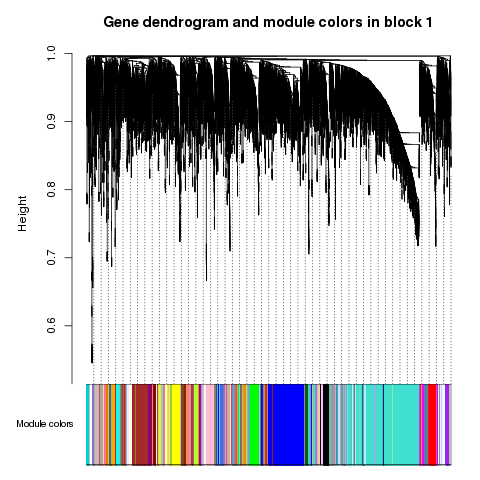


Expression Data Methylation Data

(11,374 transcripts) (14,797 transcripts)

(b)
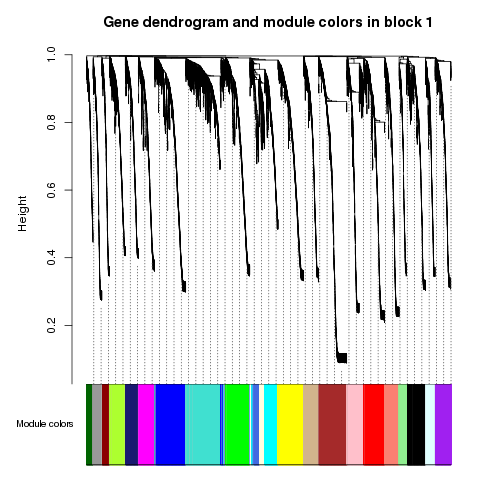

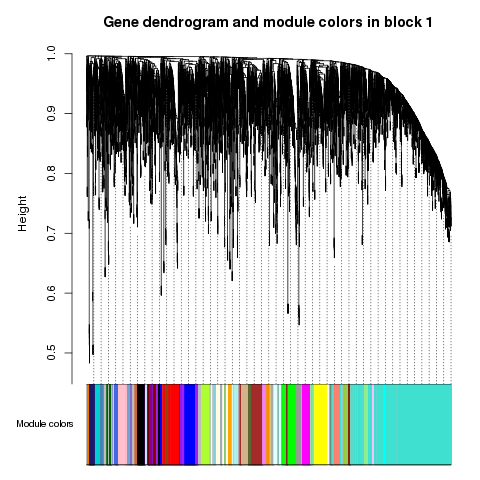


Expression Data Methylation Data

(6302 consensus transcripts) (6302 consensus transcripts)

(c)
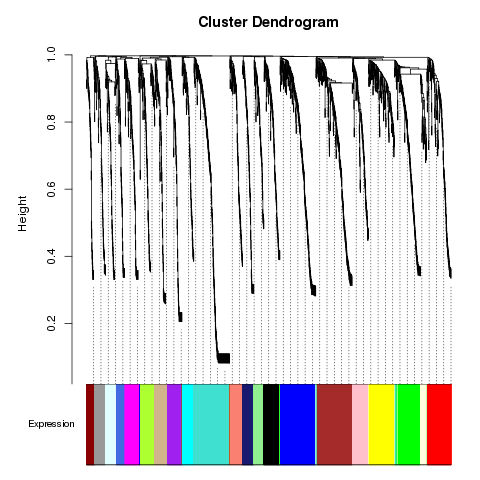

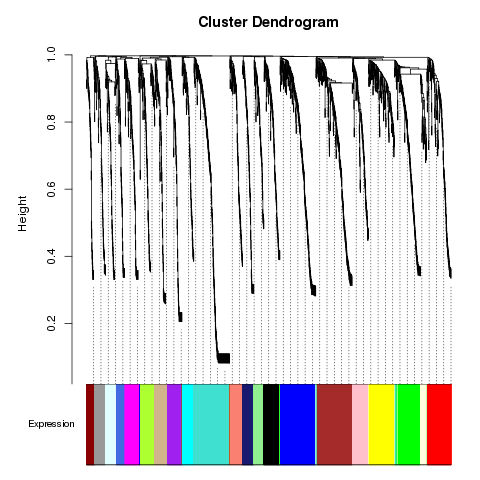


Expression Data 3’UTR Methylation Data

(1780 consensus transcripts) (1780 consensus transcripts)

**3’UTR analysis**

**Table S1: Top Hub genes**

| Module | Top hub genes |
| --- | --- |
| black | HDAC10,ECSIT,TRABD,SOX11,CHID1,RASSF6,AHDC1,NARF,ARL4C,LONRF2,WWOX,CCDC14,TBL2,AMACR,NUPL2,FKBP1B,CHL1,EXD3,NT5C2,ATXN2,FGFR1,CBX1,CDK10,CHID1,MMAB,P2RX5,RBMS2,TRAP1,DERL2,RAD51C |
| blue | ZNF282,VPS37B,ZNF710,LSM14B,PLEKHG4B,DDX39A,CCDC137,PMS1,RBCK1,ZNF521,TBC1D22A,SLC4A3,TCAIM,KIAA0930,ERC2,DMKN,DMKN,DMKN,DMKN,NADK,DCUN1D4,QDPR,LARP1,PDDC1,RNFT2,NUBPL,INO80,WWOX,TRAP1,SCPEP1 |
| brown | CBX5,CERK,SGTA,P2RX7,WDR37,SNED1,FAM109B,FOXK1,EXOC7,B4GALT2,RABEPK,GPX1,IL10RB,ITGA5,EIF4G1,CERK,DDX56,SH3BP1,DNAJC5,DPP7,CASP9,RAD51C,AKIP1,HEBP1,P2RX7,CTDSP2,ACTR10,ATP6V0D1,ATP6V0D1,TNFRSF13B |
| cyan | CATSPERG,TRAF4,ERC2,AGTPBP1,SPG7,ABLIM2,C9orf3,EPHA4,AFAP1,KYNU,LUC7L,CLTA,RUNX1,KIDINS220,ZNF566,BTNL9,DENND3,ZNF707,FOXRED1,GDPD5,CCDC91,CCND1,POLE,GABARAPL1,SCARB1,ABLIM2,CRADD,ACTR10,PPP2R5C,PSTPIP1 |
| darkred | RAB15,RGS7,THNSL2,PLEKHG5,TRABD,SNED1,USP41,C9orf3,ST3GAL3,PUM1,TMEM9,BTNL9,SPG20,EVC2,STK32B,AMACR,KCNK9,,PUM1,CTSH,ST3GAL3,GABARAPL1,TMBIM4,POC1B,DUOX1,RHOT2,NSMCE1,ENTHD2,BAIAP2,LCAT |
| green | HRK,PWWP2B,DDI2,D2HGDH,TCF20,,PFKL,OSBPL3,EEA1,ERN1,HS1BP3,ACAP2,SRPK2,DNAJB5,AGTRAP,LARP4B,ALKBH4,TRIT1,USP46,RASA2,TVP23C-CDRT4,SNTB2,APLF,CDK2AP2,ADAM15,PTS,P2RX7,DNAJB5,ANO6,GLOD4 |
| greenyellow | SP1,DFFB,LAMC3,KCNK5,ADAMTS6,DIP2C,THADA,XXYLT1,BAIAP2,LAIR1,UBE2J2,USP34,XXYLT1,RIT1,CREM,C1orf159,NDUFC1,ANKRD33B,,RAB40C,EGR3,AMBRA1,PTDSS2,PUM1,IPO7,EPHA10,HID1,CLIP1,PARN,TRPV3 |
| grey60 | CHD5,TMUB1,C20orf112,FGD4,5-Sep,5-Sep,HES6,GPT2,CLPB,FAM109A,5-Sep,DMKN,ZNF212,ASAP3,FGD4,MICAL3,NOP16,DTNBP1,CDK18,ASAP1,QRICH2,GEMIN2,TAF6L,DYNC2H1,PAPLN,ZNF839,HERC2,RHOT2,CCDC137,FDXR |
| lightcyan | B4GALNT3,AGBL3,TRPM2,SLC16A8,ADAM11,NVL,LMNA,RPS6KA1,IFT88,TRPM2,COLEC11,C7orf26,DBNL,PMPCA,FAM73B,HDAC10,TTC23,BRCA1,SLC37A3,MMEL1,C6orf165,CDK10,MAEA,CORO1B,SLC25A29,PBX4,MAPK8IP3,AATK,TRPV3,CAMTA2 |
| lightgreen | NDUFS7,AP1S3,STAT5A,TBC1D22A,CLIP1,RPL35,UEVLD,UNC13B,DTNB,WDFY4,YIPF4,FRMD5,FRMD5,MYLK,IRF7,CORO6,CCNL2,CCNL2,NDUFS4,DTNBP1,INTS9,PPP1R16A,SSSCA1,FNTA,SLIRP,CERS4,INO80E,NUBP2,TANC2,GRB7 |
| lightyellow | CDC34,SUPT6H,WDR5,FPGS,QSOX1,LFNG,OSBPL3,PSD4,EXOC6B,DBNL,HS1BP3,DNAJB12,MEF2D,SRPK2,TKT,DBNL,PHYKPL,STK35,GOLPH3,FAM193B,BSDC1,RBL1,RNF115,PTAFR,LATS1,SART3,TRAF3,SNAPC5,TEX14,SKAP1 |
| magenta | SECTM1,ARC,FLNB,MUC4,SLC35B2,BAD,INTS1,AACS,GIGYF2,MBOAT7,TUBGCP6,MUC4,ABCB8,ABCB8,MUC4,RFC2,OARD1,EML3,MSANTD1,PPAP2A,MSANTD1,MAP1B,BMP1,SERGEF,SERGEF,ACER3,ZNF707,SLIRP,MTHFS,SLC39A11 |
| midnightblue | MCHR1,WHSC1,MAGI1,PAX2,PAX2,LAIR1,TNFRSF8,PAX2,FCHSD1,NWD1,POR,RAB3A,B3GALNT2,TRIT1,WDR19,SMUG1,ACSF2,PABPC1,C11orf73,TASP1,VDR,SNX6,PDE8A,ANPEP,RGL3,RGL3,PLEKHG4,KIAA0513,ADCY9,P4HB |
| pink | CDC34,LYL1,SLC9A3,GNB1L,PRDM16,MAPK9,LFNG,HES6,DBNL,MAP4K3,SLC25A20,DHX30,PRDM16,HIC2,ASCC2,DNAJB12,DCUN1D4,CDK10,LCORL,MAPKBP1,RPS6KA4,ILVBL,ST3GAL3,WDR73,ATP6V0D1,ATP6V0D1,E2F4,TANGO6,LLGL2,SKAP1 |
| purple | PALD1,TRPV3,GFRA2,CCDC13,MMP14,PLD5,TMEM184A,NF2,GADD45G,EPHA10,ARHGAP23,LASP1,PLXNB1,ZFP64,ST3GAL3,MCF2L2,SLC4A11,TRIM11,RNF212,RNF212,RNF212,PEX10,THAP8,NAPRT1,PRMT3,ZNF707,PSMA8,ACTR10,PXMP2,CERS4 |
| red | MNT,ACVR1B,TUBB8,WDR33,DGAT1,TUBB8,CAPN10,GTDC1,E2F7,TXN2,KIF15,SUV420H2,ASF1B,DMKN,PET112,C5orf63,SPATA20,PDGFRB,RHOBTB2,NDUFS8,PSMD9,RASA3,HMBS,ETFA,MVD,ULK3,ULK3,VAC14,NUBP2,VAC14 |
| royalblue | HELLS,HYAL1,ORAOV1,ZNF534,HYAL1,SETD2,ASCL2,DNMT3A,MAPK9,DCLK2,NHSL1,LIMK1,5-Sep,UQCC1,UBE2J2,DHX35,HCK,ZWINT,EXD3,TSPAN17,PSD3,ZFAT,,PTK2,ZNF707,INPPL1,AREL1,CASC1,AKAP1,SPNS3 |
| salmon | UPF1,MXRA8,C17orf107,ODF2,ST3GAL2,IL15RA,CDC14B,NDUFA10,BPHL,ERCC3,GDPD5,IL15RA,THNSL2,STAT5A,ANKMY2,PEMT,ST3GAL3,ABCA2,GK,ST3GAL3,CREM,NSMCE2,SLC43A1,ZNF517,IL15RA,ST3GAL3,TGFBR3,MGRN1,FLII,PEMT |
| tan | DCST1,JAKMIP3,MORN1,AMACR,TBC1D7,TRAPPC10,CAPN10,TRAPPC12,WDR60,CAMK2G,RADIL,ABCA2,PDDC1,RFC2,ADAMTS13,PARK2,TTC23,BMP1,UBE2G2,TRIT1,ADAMTS13,PRKRIP1,,GLRX,SGCA,ZNF707,CHID1,SCARF1,FDXR |
| turquoise | C1orf21,PODN,GREM2,VENTX,PODN,C10orf105,ABLIM2,GLT1D1,C10orf105,CDH23,TNFRSF25,CCDC154,ABCC3,KIAA1324,DAGLA,SCARB1,ATP10A,TNFAIP2,GPR56,MTHFSD,CUEDC1,ACE |
| yellow | POLR2E,CDKN1B,BTBD2,CHST12,C8orf82,DEXI,EHD1,AP1S3,PIK3R2,CYP20A1,RPL28,DGCR14,PHF20,CDKN1B,SETD2,ICMT,ICMT,TBC1D10B,MYO1G,MXD4,MTDH,SSSCA1,RNH1,SWAP70,RNF115,INO80E,CDIPT,HGS,SKAP1,LMNB2 |

**Table S2: Expression module table for 3’UTR WGNA analysis**

| **Expression Module** | **Count** | **Term** | **Count** | **P-Value** |
| --- | --- | --- | --- | --- |
| Turquoise | 181 | TNFR/CD27/30/40/95 cysteine-rich region | 5 | 9.21E-05 |
|  |  | leukocyte proliferation | 4 | 3.94E-03 |
|  |  | cell adhesion | 13 | 4.59E-03 |
|  |  | synaptic vesicle exocytosis | 3 | 5.13E-03 |
|  |  | Apoptosis/ cell death | 13 | 5.67E-03 |
|  |  | positive regulation of secretion | 5 | 8.07E-03 |
|  |  | glycoprotein | 44 | 8.42E-03 |
|  |  | ribonucleotide binding | 24 | 1.13E-02 |
| Blue | 176 | GTPase regulator activity | 13 | 2.60E-04 |
|  |  | regulation of Rho/Ras protein signal transduction | 9 | 4.73E-04 |
|  |  | mutagenesis site | 31 | 5.34E-04 |
|  |  | mTOR signaling pathway | 4 | 8.75E-03 |
| Brown | 172 | Apoptosis/cell death | 17 | 1.60E-04 |
|  |  | phosphoprotein | 74 | 7.94E-04 |
| Yellow | 125 | compositionally biased region:Gln-rich | 5 | 7.89E-03 |
|  |  | protein domain specific binding | 7 | 1.45E-02 |
|  |  | Rab-GAP TBC | 3 | 2.09E-02 |
|  |  | regulation of Rab protein signal transduction | 3 | 2.66E-02 |
|  |  | negative regulation of epidermal growth factor receptor signaling pathway | 2 | 3.20E-02 |
|  |  | Phosphatidylinositol signaling system | 3 | 3.35E-02 |
|  |  | phosphoprotein | 48 | 3.65E-02 |
| Green | 121 | Folate biosynthesis | 3 | 1.56E-03 |
|  |  | Heat shock protein DnaJ | 3 | 8.02E-03 |
|  |  | zinc ion binding | 20 | 3.90E-02 |
|  |  | Mitochondrial carrier protein | 3 | 1.01E-02 |
|  |  | Antiviral defense | 3 | 4.36E-02 |
|  |  | transcription factor | 3 | 4.75E-02 |
| Red | 116 | nucleotide binding | 29 | 8.07E-06 |
|  |  | kinase | 11 | 2.10E-03 |
|  |  | ATP binding | 18 | 2.11E-03 |
|  |  | phosphoprotein | 49 | 7.43E-03 |
| Black | 79 | nucleotide binding | 20 | 3.06E-03 |
|  |  | phosphoprotein | 39 | 8.87E-03 |
|  |  | Protein kinase, ATP binding site | 6 | 4.23E-02 |
| Pink | 79 | repeat:WD 3 | 6 | 2.53E-03 |
|  |  | WD40 repeat | 6 | 2.87E-03 |
|  |  | activator | 7 | 1.01E-02 |
|  |  | phosphoprotein | 36 | 1.45E-02 |
|  |  | regulation of transcription | 18 | 1.47E-02 |
|  |  | JNK cascade | 3 | 2.16E-02 |
|  |  | stress-activated protein kinase signaling pathway | 3 | 2.44E-02 |
|  |  | transcription factor binding | 6 | 4.66E-02 |
| Magenta | 72 | regulation of protein kinase cascade | 5 | 9.98E-03 |
|  |  | phosphoprotein | 29 | 4.12E-02 |
|  |  | positive regulation of I-kappaB kinase/NF-kappaB cascade | 3 | 4.29E-02 |
| Purple | 71 | zinc ion binding | 16 | 1.36E-03 |
|  |  | negative regulation of cell-matrix adhesion | 2 | 2.92E-02 |
|  |  | proteolysis | 8 | 3.31E-02 |
|  |  | cofactor biosynthetic process | 3 | 3.33E-02 |
|  |  | Pleckstrin homology | 4 | 6.07E-02 |
| Greenyellow | 71 | mutagenesis site | 15 | 5.11E-03 |
|  |  | Ras | 4 | 8.56E-03 |
|  |  | mRNA 3’-UTR binding | 2 | 3.91E-02 |
|  |  | nucleotide-binding | 11 | 4.22E-02 |
|  |  | posttranscriptional regulation of gene expression | 4 | 4.73E-02 |
|  |  | compositionally biased region:Ser-rich | 5 | 4.91E-02 |
|  |  | RNA polymerase II transcription factor activity | 4 | 5.81E-02 |
| Tan | 63 | steroid metabolic process | 4 | 1.76E-02 |
|  |  | h_parkinPathway:Role of Parkin in the Ubiquitin-Proteasomal Pathway | 2 | 1.87E-02 |
| Salmon | 60 | mitochondrion | 11 | 1.51E-03 |
|  |  | organelle membrane | 11 | 1.61E-03 |
|  |  | mutagenesis site | 13 | 7.67E-03 |
|  |  | ATPase activity, coupled | 5 | 8.71E-03 |
|  |  | Restriction endonuclease, type I, R subunit/Type III, Res subunit | 2 | 1.15E-02 |
|  |  | regulation of activated T cell proliferation | 2 | 3.84E-02 |
| Cyan | 57 | androgen metabolic process | 2 | 3.44E-02 |
|  |  | nucleotide binding | 14 | 1.75E-02 |
|  |  | zinc ion binding | 13 | 4.90E-02 |
| Midnightblue | 55 | protein C-terminus binding | 5 | 2.56E-04 |
|  |  | extrinsic to membrane | 6 | 5.41E-03 |
|  |  | protein transport/ establishment of protein localization | 7 | 1.43E-02 |
|  |  | compositionally biased region: Poly-Ala | 5 | 1.57E-02 |
|  |  | nucleotide binding | 11 | 1.96E-02 |
| Grey60 | 52 | Cell division and chromosome partitioning / Cytoskeleton | 3 | 4.22E-03 |
|  |  | ion binding and zinc ion binding | 18 | 1.32E-02 |
|  |  | regulation of Ras GTPase activity | 3 | 2.08E-02 |
|  |  | Pleckstrin homology-type | 4 | 3.36E-02 |
| Lightcyan | 52 | phosphorylation | 6 | 3.81E-02 |
|  |  | binding site:ATP | 5 | 4.09E-02 |
|  |  | chordate embryonic development | 4 | 4.27E-02 |
|  |  | transmembrane transport | 5 | 4.39E-02 |
|  |  | smoothened signaling pathway | 2 | 4.40E-02 |
|  |  | cytoskeletal protein binding | 5 | 4.54E-02 |
|  |  | Cell Cycle: G2/M Checkpoint | 2 | 4.73E-02 |
| Lightyellow | 45 | Apoptosis | 4 | 4.73E-02 |
|  |  | protein kinase binding | 3 | 4.78E-02 |
|  |  | Transcription | 9 | 6.79E-02 |
| Royalblue | 42 | nucleotide-binding | 13 | 2.44E-05 |
|  |  | kinase | 7 | 1.37E-03 |
|  |  | methylation-dependent chromatin silencing | 2 | 1.03E-02 |
|  |  | methylation | 3 | 1.07E-02 |
|  |  | protein amino acid phosphorylation | 6 | 1.10E-02 |
|  |  | focal adhesion | 3 | 1.43E-02 |
| Darkred | 41 | vacuole | 3 | 6.36E-02 |

**Table S3: Methylation module table for 3’UTR WGNA analysis**

| **Module** |  | **Term** | **Count** | **P-value** |
| --- | --- | --- | --- | --- |
| Turquoise | 284 | DNA repair | 10 | 5.23E-03 |
|  |  | folic acid and derivative biosynthetic process | 3 | 5.46E-03 |
|  |  | acetylation | 42 | 8.99E-03 |
|  |  | vesicle-mediated transport | 14 | 1.44E-02 |
|  |  | Histone core | 4 | 1.50E-02 |
|  |  | cell cycle checkpoint | 5 | 2.01E-02 |
|  |  | membrane organization | 10 | 3.03E-02 |
|  |  | DNA binding | 9 | 3.15E-02 |
|  |  | SOX-12/11/4a protein | 2 | 3.25E-02 |
|  |  | intracellular organelle lumen | 29 | 3.40E-02 |
|  |  | nucleotide-binding | 27 | 3.97E-02 |
|  |  | repeat: WD 5 | 7 | 4.16E-02 |
|  |  | Apoptosis | 9 | 5.44E-02 |
| blue | 224 | phosphoprotein | 71 | 5.74E-04 |
|  |  | regulation of cellular protein metabolic process | 12 | 1.11E-03 |
|  |  | negative regulation of transferase activity | 5 | 6.59E-03 |
|  |  | negative regulation of cell proliferation | 9 | 7.02E-03 |
|  |  | regulation of protein modification process | 8 | 8.07E-03 |
|  |  | mutagenesis site | 23 | 2.95E-02 |
| brown | 197 | regulation of hydrolase activity | 9 | 3.48E-03 |
|  |  | regulation of peptidase activity | 4 | 2.58E-02 |
|  |  | lipid localization | 5 | 2.90E-02 |
|  |  | protein complex assembly | 9 | 3.33E-02 |
|  |  | regulation of Ras/Rab GTPase activity | 4 | 4.18E-02 |
| Yellow | 165 | Focal adhesion | 9 | 3.88E-05 |
|  |  | nucleotide-binding | 23 | 1.47E-04 |
|  |  | kinase | 13 | 4.82E-04 |
|  |  | transferase | 17 | 4.77E-03 |
|  |  | phosphoprotein | 56 | 3.53E-03 |
|  |  | plasma membrane | 31 | 1.87E-02 |
|  |  | Proton acceptor | 10 | 1.23E-02 |
| green | 132 | compositionally biased region: Pro-rich | 14 | 1.87E-04 |
|  |  | hydrolase | 14 | 1.27E-02 |
|  |  | positive regulation of apoptosis | 7 | 1.36E-02 |
|  |  | nucleoside binding | 14 | 3.87E-02 |
| red | 101 | ligase activity, forming carbon-nitrogen bonds | 6 | 1.29E-03 |
|  |  | Regulator of chromosome condensation/beta-lactamase-inhibitor protein II | 3 | 1.82E-03 |
|  |  | GTPase regulator activity | 7 | 2.86E-03 |
| black | 96 | phospholipid binding | 6 | 8.92E-04 |
|  |  | cell projection | 8 | 1.03E-02 |
|  |  | phosphoprotein | 35 | 1.56E-02 |
| pink | 93 | nucleotide-binding | 15 | 6.58E-04 |
|  |  | transferase | 10 | 3.08E-02 |
|  |  | phosphorylation | 8 | 3.24E-02 |
|  |  | metal ion-binding site:Iron-sulfur (4Fe-4S) | 2 | 4.99E-02 |
| Magenta | 79 | regulation of Ras protein signal transduction | 4 | 1.31E-02 |
|  |  | transcription | 11 | 1.95E-02 |
|  |  | acetylation | 12 | 3.71E-02 |
|  |  | WD40/YVTN repeat-like | 4 | 3.73E-02 |
| purple | 68 | RNA processing | 6 | 8.65E-03 |
|  |  | regulation of Ras protein signal transduction | 4 | 1.31E-02 |
|  |  | transcription | 11 | 1.95E-02 |
|  |  | domain:Ig-like C2-type | 3 | 2.45E-02 |
|  |  | acetylation | 12 | 3.71E-02 |
|  |  | IPR015943:WD40/YVTN repeat-like | 4 | 3.73E-02 |
| greenyellow | 56 | transferase | 11 | 9.87E-04 |
|  |  | mutagenesis site | 13 | 1.75E-03 |
|  |  | nucleotide-binding | 11 | 4.06E-03 |
|  |  | metal-binding | 15 | 5.03E-03 |
|  |  | cell junction | 5 | 1.29E-02 |
| tan | 56 | ribonucleotide binding | 9 | 7.95E-03 |
|  |  | regulation of Rho/Ras protein signal transduction | 3 | 1.21E-02 |
| salmon | 53 | phosphoprotein | 22 | 3.84E-03 |
|  |  | exonuclease activity | 3 | 4.26E-03 |
|  |  | ADP-ribosylation | 2 | 5.65E-02 |
|  |  | transcription coactivator activity | 3 | 5.89E-02 |
|  |  | cytoskeletal protein binding | 4 | 6.43E-02 |
| Cyan | 48 | s-adenosyl-l-methionine | 3 | 1.27E-02 |
|  |  | metalloprotease | 3 | 2.60E-02 |
| Midnightblue | 45 | Peptidase M, neutral zinc metallopeptidases, zinc-binding site | 3 | 7.04E-03 |
|  |  | 4 iron, 4 sulfur cluster binding | 2 | 2.66E-02 |
|  |  | cofactor binding | 3 | 2.86E-02 |
|  |  | ion binding | 9 | 5.13E-02 |
| Lightcyan | 44 | zinc ion binding | 10 | 6.62E-03 |
|  |  | repressor | 4 | 2.72E-02 |
|  |  | regulation of transcription, DNA-dependent | 7 | 2.99E-02 |
|  |  | mutagenesis site | 8 | 3.00E-02 |
|  |  | SH2 domain binding | 2 | 3.81E-02 |
|  |  | T_cell_receptor | 2 | 4.47E-02 |
|  |  | histone deacetylase binding | 2 | 4.90E-02 |
|  |  | intracellular non-membrane-bounded organelle | 8 | 5.50E-02 |
|  |  | h_agrPathway:Agrin in Postsynaptic Differentiation | 2 | 5.54E-02 |
| Grey60 | 37 | compositionally biased region:Poly-Glu | 5 | 3.11E-03 |
|  |  | phosphoprotein | 16 | 1.21E-02 |

**Table S4: Significant Overlap table for 3’UTR WGCNA**

| **Methyaltion Module (gene count)** | **Expression Module (gene count)** | **P-Value** | **Gene Symbols** |
| --- | --- | --- | --- |
| Magenta (79) | Turquoise (181) | 6.17E-05 | ABCC3,ALS2CL,ATG16L2,COG8,DNASE1,DOCK9,ESPL1,PDE4D,TMEM63C,TNFAIP2,TNFRSF4,TTLL1,UQCC1,ZNF385A |
| Red (101) | grey60 (52) | 2.04E-03 | CHID1,FDXR,HERC2,HERC6,MYO9B,SEPT5,UBXN7 |
| Yellow (165) | Turquoise (181) | 3.18E-03 | ATG16L2,BMP1,C1orf159,CLUH,COL6A2,CRTC1,DAGLA,DNAH3,ENTHD2,EPHA10,FAM101B,FAM53A,GPR56,IGF1R,LAMC3,NLRP2,NR3C2,OPLAH,PITPNM2,PPP2R2B,PRKCA,PTPRK,RASGRF1,STK32C,THNSL2 |
| Turquoise (284) | Blue (176) | 4.83E-03 | ACOXL,ANKH,ARHGEF18,B3GAT3,CCAR1,CCDC137,CKB,CREM,DDX39A,DFFB,DNAJB12,FAIM3,HUS1,ITPK1,KIAA0930,LRPAP1,MGRN1,MRPS24,NADK,NRARP,NUBPL,NUP155,OSBPL7,PHF14,PIP5K1A,PPFIA3,PSTPIP1,PTMA,RALBP1,RIN3,RPS6KB2,SH3BP2,SH3TC1,TP53I3,TRAF4,TRIB2,TSSC1,USP42 |
| Brown (197) | Lightgreen (49) | 5.48E-03 | UEVLD,UNC13B,DTNB,BANP,IRF7,CCNL2,INTS9,FNTA,RBM19,DNASE1,GRB7 |
| Midnightblue (45) | Turquoise (181) | 1.24E-02 | ABLIM2,ACE,COMMD1,ELFN2,GLT1D1,HAUS7,TOM1 |
| Turquoise (284) | Midnightblue (55) | 2.11E-02 | ADCY9,AGTRAP,BSDC1,INO80E,MCHR1,MTHFSD,NADK,P4HB,PDE8A,POR,RAB3A,RPS19BP1,SMUG1,SOX12,STX8 |
| Greenyellow (56) | Greenyellow (71) | 2.18E-02 | C9orf3,DIP2C,KCNK5,NCLN,SH3BP5,TOR3A |
| Lightcyan (44) | Green (121) | 2.63E-02 | CCNF,GABARAPL1,TBC1D14,USP46,ZNF589 |
| Blue (224) | Purple (71) | 2.68E-02 | ACTR10,BRE,DMKN,FAM109A,GPR137,IL17RC,MCF2L2,NF2,ST3GAL3,TRPV3,ZFP64 |
| Black (96) | Salmon (60) | 3.94E-02 | DBNL,TGFBR3 |
| Purple (69) | Green (121) | 4.05E-02 | ACAP2,BRD9,DNAJB5,FAM107B,GTF3C1,THOC3,TRIT1 |
| Black (96) | Blue (176) | 4.55E-02 | ADAT3,AKT2,INO80,ITPR2,JMJD7PLA2G4B,LSM1,PMS1,POC1B,POLR3A,RBCK1,SCPEP1,SLC22A5,VDR,ZNF282 |
| Midnightblue (45) | Pink (79) | 4.62E-02 | ADAM15,ATP6V0D1, LLGL2 |
| Blue (224) | Lightcyan (52) | 5.37E-02 | C9orf89,DBNL,FAM73B,FN3KRP,IFT88,KANSL3,NVL,PET112,SLC25A29,SLC37A3,TMEM184B |
| Red (101) | Lightgreen (49) | 5.54E-02 | FRMD5,GAK,HERC4,SYNGR1,YIPF4 |

**Table S5: Correlation table for 3’UTR WGCNA**

| **Methylation Module** | **Expression Module** | **Correlation** | **P-Value** |
| --- | --- | --- | --- |
| **Negative Correlation** | | | |
| red | black | -0.8695127 | 1.34E-06 |
| purple | blue | -0.8661847 | 1.64E-06 |
| cyan | cyan | -0.9033185 | 1.18E-07 |
| black | greenyellow | -0.8992664 | 1.64E-07 |
| pink | grey60 | -0.9016512 | 1.35E-07 |
| grey60 | magenta | -0.9274494 | 1.11E-08 |
| green | red | -0.9418253 | 1.79E-09 |
| brown | salmon | -0.9187598 | 2.83E-08 |
| greenyellow | tan | -0.9118115 | 5.55E-08 |
| blue | turquoise | -0.9156028 | 3.87E-08 |
| **Positive Correlation** | | | |
| midnightblue | red | 0.5035422 | 0.02795393 |
| tan | red | 0.4884827 | 0.03382908 |

**Non-Coding gene analysis**

**Table S6: Non-coding Expression module top gene**

| Modules | Top 10 Module Genes |
| --- | --- |
| Lightcyan | RGS7, PLEKHG5, NLRP2, SNED1, USP41, CYP2J2, BUB1, CYP2J2, PDHB, USP41 |
| Greenyellow | SOX11, AHDC1, ARL4C, LONRF2, CHL1, CCDC14, LCN8, FGFR1, EGR2, PHF21A |
| Brown | ABLIM2, VENTX, FEZ1, C10orf105, CDH23, TLL2, SH2D2A, EBF1, KIAA1324, SCARB1 |
| Darkgreen | C17orf107, IL15RA, CDC14B, IL15RA, STAT5A, PEMT, ST3GAL3, SMYD3, C3orf17, URGCP |
| Royalblue | CHD5, EPB41L4A, C20orf112, FGD4, CLPB, ARHGAP22, PFN4, AASS, SPTLC1, DAGLB |
| Lightyellow | B4GALNT3, AGBL3, SLC16A8, TRPM2, COLEC11, ELP6, ULK4, COL9A2, UBAP2, GLB1 |
| Grey60 | HYAL1, ZNF534, MLLT4-AS1, NHSL1, LPAL2, SIAE, TNFRSF14, ZWINT, AKT2, CKMT2-AS1 |
| Magenta | ANKRD20A11P, MUC4, FAM65C, ZNF442, TLE1, MUC4, COMTD1, ZNF717, BMP1, GPATCH4 |
| Pink | PAX2, YARS, PLA2G2C, PABPC1, ST3GAL1, VDR, ANXA2, LOXL1-AS1, RGL3, ADCY9 |
| Turquoise | POLR2E, DEXI, PSPC1P1, SBF1P1, NDUFA5, CALM2, RPL18P11, PAPSS1, LYPLA1, SSSCA1 |
| Tan | PALD1, DCST2, EPHA10, PCOLCE-AS1, UQCC1, APBB2, PEX10, PRMT3, ALOX12B, LINC00426 |
| Red | AMACR, NFIX, C11orf95, ABCA2, UBAP2, NT5DC3, ABCA2, C1orf159, RUNX3, CAMK2G |
| Black | EPHA4, LINC00163, ZNF3, CLTA, GLB1, ZNF566, EBF1, ZNF707, PRR4, SCARB1 |
| Midnightblue | SLC37A3, TMEM120A, PRKAB2, AKT1S1, RAB40C, GDPD5, CARS2, POLE, TIMM9, LOXL1-AS1 |
| Cyan | AMBRA1, LINC00324, UNC13B, FRMD5, SIT1, TMCO4, ESYT2, TNRC18P1, TRIQK, GANAB |
| Green | CBX5, CERK, SGTA, WDR37, XRCC6P1, LINC00959, ANAPC1, FAM194B, EIF4G2, C14orf37 |
| Yellow | PWWP2B, TCF20, DNAJB5, ELMO2, KDM5B, CACNA2D2, RASA2, TVP23C-CDRT4, CDK2AP2, P2RX7 |
| Salmon | SLC9A3, MTUS2-AS1, SLC23A2, MAPK9, HES6, DCST1, TNKS1BP1, MAP4K3, PPAP2B, TAF5L |
| Darkred | WDR5, OSBPL3, C9orf114, ATP13A1, KCNH2, NADK, USF1, GOLPH3, H2AFZ, STK10 |
| Lightgreen | SUPT6H, QSOX1, PSD4, EXOC6B, EIF4E, ADAR, HS6ST1, GNA12, AIDA, ENO1 |
| Blue | VPS37B, LSM14B, CCDC137, HELZ2, DMKN, DUSP11, MYO1G, MACROD1, UEVLD, GPN3 |
| Purple | MNT, DDX12P, WDR33, TUBB8, KIF15, MIR4435-1HG, C5orf63, KIAA0196, RHOBTB2, BCL9L |

**Table S7: Non-coding genes expression GO annotation table**

| **Module** | **Size** | Term | **Pvalue** |
| --- | --- | --- | --- |
| **turquoise** | 627 | RNA processing/ Negative regulation of cellular biosynthetic process/transcription | 0.006 |
|  |  | Translation | 0.013 |
|  |  | Regulation of Rab protein signal transduction | 0.025 |
|  |  | Regulation of Ras protein signal transduction | 0.028 |
|  |  | Chromatin organization | 0.037 |
|  |  | Cofactor metabolic process | 0.043 |
| **blue** | 541 | Intracellular signaling cascade | 0.016 |
| **brown** | 492 | Cell death/apoptosis | 0.003 |
|  |  | Leukocyte proliferation | 0.039 |
| **yellow** | 449 | Protein localization | 0.012 |
|  |  | Positive regulation of apoptosis | 0.014 |
|  |  | Regulation of Ras protein signal transduction | 0.016 |
|  |  | Cellular macromolecule catabolic process | 0.016 |
| **green** | 420 | RNA processing | 0.004 |
|  |  | Establishment of protein localization/protein transport | 0.016 |
|  |  | Methylation | 0.044 |
| **red** | 366 | Sterol metabolic process | 0.000 |
|  |  | Endocytosis | 0.021 |
|  |  | Oxidation reduction | 0.059 |
|  |  | Positive regulation of apoptosis | 0.093 |
| **black** | 314 | Protein transport/establishment of protein localization | 0.040 |
|  |  | Cofactor/co-enzyme metabolic process | 0.001 |
|  |  | Cellular amide metabolic process | 0.000 |
|  |  | T cell differentiation | 0.034 |
|  |  | RNA 3'-end processing | 0.035 |
| **pink** | 287 | Attachment of GPI anchor to protein | 0.003 |
|  |  | Posttranscriptional regulation of gene expression | 0.032 |
|  |  | Phosphorus metabolic process | 0.046 |
|  |  | Induction of apoptosis | 0.069 |
| **magenta** | 282 | Phosphorylation | 0.000 |
| **purple** | 279 | Vesicle-mediated transport/endocytosis | 0.000 |
| **greenyellow** | 274 | Negative regulation of transcription | 0.000 |
| **tan** | 260 | Negative regulation of cell cycle | 0.012 |
|  |  | Proteolysis | 0.039 |
| **salmon** | 249 | Proteolysis involved in cellular protein catabolic process | 0.012 |
|  |  | Negative regulation of cell cycle | 0.030 |
|  |  | Negative regulation of protein kinase activity | 0.036 |
|  |  | Cell cycle arrest | 0.055 |
|  |  | Apoptosis | 0.065 |
| **cyan** | 248 | Ubiquitin-dependent protein catabolic process | 0.004 |
|  |  | Phosphorus metabolic process | 0.028 |
|  |  | Methylation | 0.044 |
| **midnightblue** | 222 | Protein transport/localization/targetting | 0.032 |
| **lightcyan** | 171 | Cell division | 0.012 |
|  |  | Phosphorylation | 0.032 |
| **grey60** | 162 | Cytoskeleton organization | 0.028 |
|  |  | T cell activation | 0.030 |
|  |  | Apoptosis/ cell death | 0.037 |
| **lightgreen** | 144 | Activation of MAPK activity | 0.002 |
|  |  | Phosphorus metabolic process | 0.004 |
|  |  | Activation of JUN kinase activity | 0.012 |
|  |  | JNK cascade | 0.055 |
| **lightyellow** | 141 | Translation | 0.008 |
| **royalblue** | 138 | Negative regulation of macromolecule metabolic process | 0.007 |
|  |  | Negative regulation of ubiquitin-protein ligase activity during mitotic cell cycle | 0.050 |
| **darkred** | 123 | Sphingolipid biosynthetic process | 0.009 |
|  |  | Intracellular transport | 0.012 |
|  |  | Regulation of Ras GTPase activity | 0.013 |
| **darkgreen** | 113 | Regulation of cell cycle | 0.020 |

**Table S8: Non-coding genes methylation GO annotation table**

Grey (Transcripts not assigned to any particular module) - 17

| **Module** | **Size** | **Term** | **PValue** |
| --- | --- | --- | --- |
| **Turquoise** | 1911 | Negative regulation of transcription | 0.000 |
|  |  | Regulation of cell death/ apoptosis | 0.001 |
|  |  | Proteasomal ubiquitin-dependent protein catabolic process | 0.001 |
|  |  | ER-nuclear signaling pathway | 0.005 |
|  |  | Mitotic cell cycle/G1/S transition of mitotic cell cycle | 0.009 |
|  |  | Regulation of Wnt receptor signaling pathway | 0.020 |
| **Blue** | 321 | Translation | 0.005 |
|  |  | Positive regulation of apoptosis | 0.009 |
|  |  | Protein transport/localization | 0.016 |
|  |  | Membrane organization | 0.032 |
| **Brown** | 291 | Actin filament organization | 0.005 |
|  |  | Macromolecular complex subunit organization | 0.007 |
|  |  | Regulation of GTPase activity | 0.029 |
|  |  | Negative regulation of cell cycle | 0.041 |
|  |  | Regulation of protein localization | 0.042 |
|  |  | Regulation of Ras protein signal transduction | 0.049 |
|  |  | Rho protein signal transduction | 0.050 |
| **Yellow** | 278 | Cell-matrix adhesion | 0.033 |
|  |  | Phosphoinositide biosynthetic process | 0.040 |
|  |  | Regulation of small GTPase mediated signal transduction | 0.050 |
| **Green** | 277 | Activation of MAPKKK cascade | 0.006 |
| **Red** | 272 | regulation of cell cycle | 0.003 |
|  |  | Intracellular signaling cascade | 0.019 |
| **Black** | 205 | Macromolecule catabolic process | 0.002 |
|  |  | Negative regulation of cell cycle | 0.013 |
| **Pink** | 171 | Phosphorylation | 0.001 |
| **Magenta** | 165 | Regulation of transcription | 0.008 |
| **Purple** | 161 | protein localization | 0.005 |
| **Greenyellow** | 155 | Cell death/apoptosis | 0.019 |
| **Tan** | 133 | Steroid metabolic process | 0.006 |
| **Salmon*** | 122 | Definitive hemopoiesis | 0.027 |
|  |  | Lymphocyte activation | 0.041 |
| **Cyan*** | 119 | Translational elongation | 0.052 |
| **Midnightblue** | 115 | Cellular macromolecule catabolic process | 0.013 |
| **Lightcyan*** | 115 | C-terminal protein amino acid methylation | 0.007 |
| **Grey60*** | 114 | Response to lipopolysaccharide | 0.004 |
| **Lightgreen** | 99 | Regulation of protein ubiquitination | 0.026 |
|  |  | Macromolecule catabolic process | 0.044 |
|  |  | Enzyme linked receptor protein signaling pathway | 0.054 |
| **Lightyellow*** | 92 | Phosphorus metabolic process | 0.085 |
| **Royalblue** | 85 | Regulation of lipid metabolic process | 0.034 |
| **Darkred** | 84 | RNA processing | 0.031 |
| **Darkgreen** | 75 | Activation of MAPKKK cascade | 0.006 |
| **Darkturquoise** | 74 | Establishment of cell polarity | 0.036 |
| **Darkgrey** | 73 | Activation of caspase activity | 0.006 |
| **Orange*** | 72 | Glutamate signaling pathway | 0.045 |
|  |  | Ion transport | 0.050 |
| **Darkorange*** | 71 | Microtubule cytoskeleton organization | 0.037 |
| **Skyblue*** | 68 | M phase | 0.022 |
| **Saddlebrown*** | 62 | mRNA metabolic process | 0.024 |
| **Steelblue** | 61 | ncRNA processing | 0.040 |
| **Violet** | 60 | Protein localization | 0.006 |
| **Paleturquoise** | 60 | ATP biosynthetic process | 0.012 |
|  |  | Purine ribonucleoside triphosphate biosynthetic process | 0.014 |
| **Darkolivegreen*** | 59 | JNK cascade | 0.068 |
| **Darkmagenta** | 57 | Phosphorylation | 0.008 |
| **Sienna3*** | 53 | NAD metabolic process | 0.043 |
|  |  | Regulation of ARF GTPase activity | 0.049 |
| **Yellowgreen*** | 47 | ATP biosynthetic process | 0.010 |
|  |  | Purine ribonucleoside triphosphate biosynthetic process | 0.012 |
| **Skyblue3*** | 38 | Phosphorus metabolic process | 0.030 |

**Table S9: Median Rank for methylation and expression preservation**

**Expression**

| **Module** | **Median Rank** |
| --- | --- |
| darkgreen | 2 |
| lightyellow | 3 |
| brown | 8 |
| darkred | 8 |
| pink | 9 |
| purple | 9 |
| black | 10 |
| midnightblue | 11 |
| greenyellow | 12 |
| salmon | 12 |
| red | 13 |
| yellow | 13 |
| lightcyan | 14 |
| cyan | 15 |
| tan | 15 |
| grey60 | 16 |
| lightgreen | 16 |
| turquoise | 16 |
| blue | 17 |
| magenta | 18 |
| royalblue | 18 |
| green | 19 |

**Methylation**

| **Module** | **Median Rank** |
| --- | --- |
| paleturquoise | 7 |
| yellowgreen | 8 |
| royalblue | 9 |
| cyan | 10 |
| darkturquoise | 11 |
| orange | 11 |
| pink | 11 |
| darkgreen | 12 |
| steelblue | 12 |
| brown | 14 |
| lightyellow | 14 |
| skyblue3 | 14 |
| saddlebrown | 15 |
| yellow | 15 |
| darkmagenta | 17 |
| darkgrey | 18 |
| greenyellow | 18 |
| darkolivegreen | 19 |
| green | 20 |
| midnightblue | 20 |
| skyblue | 21 |
| white | 21 |
| darkorange | 22 |
| tan | 22 |
| lightcyan | 23 |
| sienna3 | 23 |
| turquoise | 23 |
| black | 24 |
| darkred | 24 |
| violet | 24 |
| blue | 26 |
| grey60 | 26 |
| purple | 26 |
| lightgreen | 27 |
| red | 29 |
| magenta | 32 |
| salmon | 34 |

**Table S10:** **Significant Overlap table for Non-coding gene WGCNA**

| **Expression Modules** | **Methylation Modules** | **Overlap Genes** | **P-Value** | **Enriched** **Biological Process** |
| --- | --- | --- | --- | --- |
| Blue | Yellow | 40 | 7.03E-04 | Signaling |
| Brown | Green | 37 | 7.72E-04 | MAPkk |
| Pink | Light Yellow | 12 | 8.73E-04 | Phosphorous |
| Turqoise | Turqoise | 225 | 9.40E-04 | Negative Regulation of Transcription |
| Royalblue | Turquoise | 58 | 2.12E-03 | Mitotic Cell Cycle |
| Light Cyan | Brown | 16 | 5.36E-03 | Cell Cycle |
| GreenYellow | Magenta | 14 | 1.23E-02 | Negative Regulation of Transcription |
| Light Yellow | Blue | 13 | 2.69E-02 | Translation |
| Black | Blue | 23 | 4.88E-02 | Protein Localization |

**Table S11: Eigengene Correlation table**

| **Negative Correlation** | | | |
| --- | --- | --- | --- |
| **Methylation Module** | **Expression Module** | **Correlation** | **P-Value** |
| red | brown | -0.97073 | 5.75E-12 |
| yellowgreen | cyan | -0.94104 | 2.00E-09 |
| brown | darkgreen | -0.94630 | 9.21E-10 |
| royalblue | green | -0.94129 | 1.93E-09 |
| tan | greenyellow | -0.91703 | 3.36E-08 |
| sienna3 | grey60 | -0.88337 | 5.40E-07 |
| skyblue3 | lightgreen | -0.94743 | 7.72E-10 |
| black | magenta | -0.95885 | 1.00E-10 |
| grey60 | pink | -0.93033 | 7.97E-09 |
| pink | red | -0.92005 | 2.48E-08 |
| salmon | tan | -0.95148 | 3.96E-10 |
| Green | red | -0.52885 | 0.01991 |

| **Positive Correlation** | | | |
| --- | --- | --- | --- |
| **Methylation Module** | **Expression Module** | **Correlation** | **P-Value** |
| darkolivegreen | greenyellow | 0.54677 | 0.01541 |
| magenta | blue | 0.91830 | 2.96E-08 |
| blue | darkred | 0.94448 | 1.22E-09 |
| midnightblue | grey60 | 0.54861 | 0.015 |
| lightyellow | lightcyan | 0.90749 | 8.21E-08 |
| midnightblue | lightgreen | 0.63093 | 0.003772 |
| grey | midnightblue | 0.74200 | 0.000276 |
| purple | purple | 0.89193 | 2.91E-07 |
| darkgrey | turquoise | 0.55534 | 0.01357 |
| lightcyan | turquoise | 0.55300 | 0.01406 |
| skyblue | turquoise | 0.53797 | 0.01751 |
| lightgreen | yellow | 0.90265 | 1.24E-07 |

**Figure S4: Cross-tabulation overlap table. Correspondence of methylation set-speciﬁc modules and the methylation-expression consensus modules.**


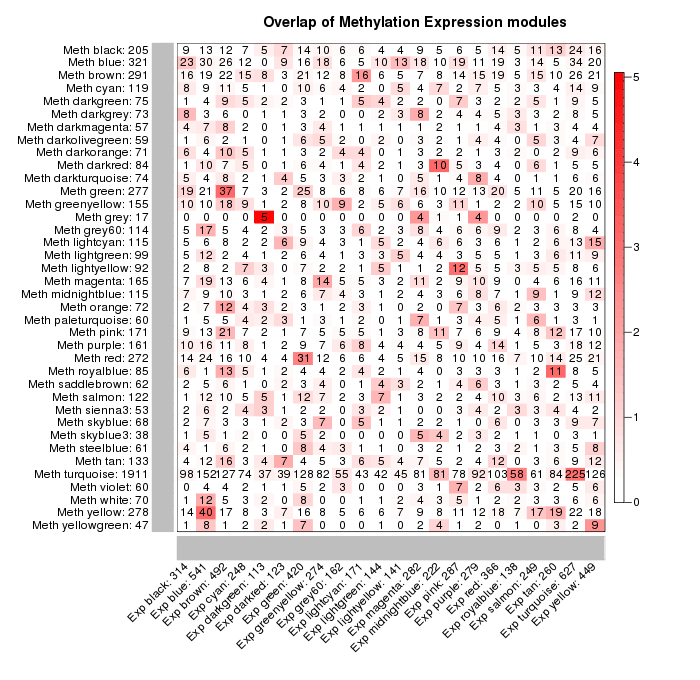


**Figure S5: The medianRank and Z_summary_ statistics of module preservation of methylation modules in non-coding gene modules (y-axis) vs. module size (x-axis).**


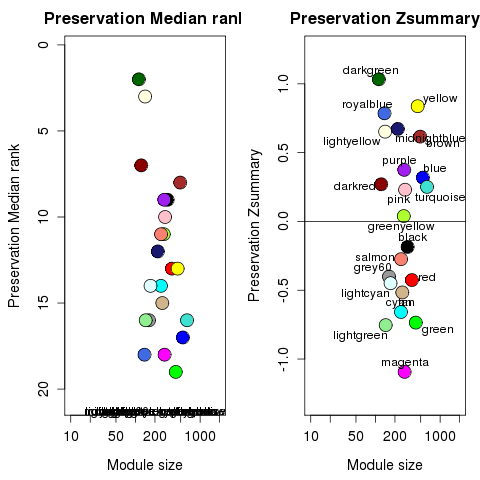


**Figure S6: Correlation of methylation and expression module eigengenes for whole non-coding genes.**

**
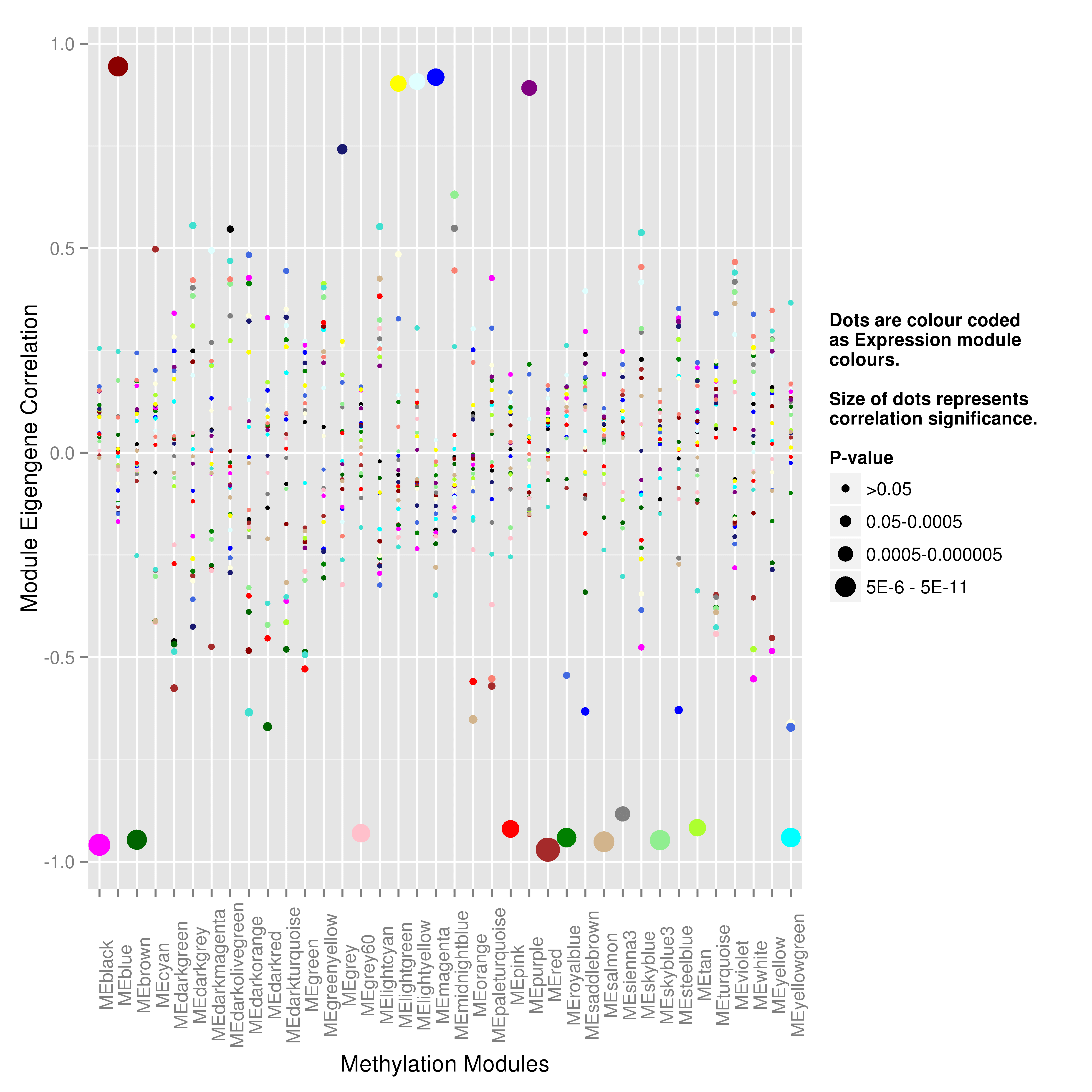
**
