# Supplementary material for: Hypomethylation coordinates antagonistically with hypermethylation in cancer development: a case study of leukemia
Source: Hum Genomics. 2016 Jul 25;10(Suppl 2):18. doi: 10.1186/s40246-016-0071-5 (PMC4965721; doi:10.1186/s40246-016-0071-5)
Supplement: Additional file 8: — Read mapping details. This file provides the mapping statistics for all the samples in both RRBS and RNA-seq datasets. Table S1 lists the number of RRBS reads obtained, their % that is uniquely mapped on human genome (hg19) along with total number of CpGs analyzed from each sample. Table S2 lists the number of RNA-seq reads obtained and their % that is uniquely mapped on human genome (hg19) for each sample. (DOCX 124 kb) [file 40246_2016_71_MOESM8_ESM.docx]

**Table S1: RRBS mapping details**

| **Number** | **Sample** | **Number of reads** | **Mapping rate** | **CpGs mapped** |
| --- | --- | --- | --- | --- |
|  | CLL030701 | 30135003 | 66.4% | 1,576,530 |
|  | CLL030801 | 23985525 | 68.7% | 1,505,794 |
|  | CLL031001 | 29136951 | 69.9% | 1,588,893 |
|  | CLL121709 | 29873514 | 70.3% | 1,702,014 |
|  | CLL131301 | 27730219 | 68.1% | 1,651,499 |
|  | CLL131401 | 28678310 | 69.6% | 1,528,890 |
|  | CLL131601 | 31454937 | 68.7% | 1,563,820 |
|  | CLL275 | 19202715 | 46.5% | 1,612,314 |
|  | CLL321 | 20092706 | 44.9% | 1,634,523 |
|  | CLL335 | 25422283 | 50.9% | 2,200,331 |
|  | CLL348 | 23256643 | 39.0% | 1,550,229 |
|  | CLL377 | 20692155 | 40.5% | 1,418,464 |
|  | CLL463 | 16213955 | 44.5% | 1,352,127 |
|  | CLL515 | 22791626 | 45.6% | 1,636,485 |
|  | CLL6159 | 20060515 | 34.0% | 1,213,691 |
|  | CLL625 | 32596751 | 51.6% | 2,439,949 |
|  | CLL6536 | 24484435 | 33.2% | 1,322,828 |
|  | CLL659 | 16717231 | 48.1% | 1,553,963 |
|  | CLL683 | 16349654 | 42.3% | 1,285,594 |
|  | CLL700 | 51869150 | 46.1% | 2,625,136 |
|  | CLL775 | 23394407 | 44.0% | 1,687,093 |
|  | CLL849 | 19651280 | 46.9% | 1,717,952 |
|  | CLL8545 | 26191713 | 38.7% | 1,626,053 |
|  | CLL8919 | 19479977 | 38.0% | 1,281,551 |
|  | CLL9128 | 19150421 | 47.3% | 1,433,567 |
|  | CLL9130 | 22897865 | 39.3% | 1,642,783 |
|  | CLL9216 | 21923354 | 42.1% | 1,497,789 |
|  | CLL9281 | 25532615 | 35.0% | 1,475,502 |
|  | CLL934 | 17345341 | 47.1% | 1,530,495 |
|  | CLL949 | 18880247 | 53.0% | 1,766,597 |
|  | **Control Samples** | **Number of reads** | **Mapping rate** | **CpGs mapped** |
|  | Control/SC1 | 18733618 | 49.5% | 1,505,570 |
|  | Control/SC2 | 13402805 | 45.8% | 1,024,275 |
|  | Control/SC4_1 | 42615706 | 34.1% | 2,013,134 |
|  |  |  |  |  |

**Table S2: Rnaseq data mapping details**

| **Number** | **Sample** | **Number of reads** | **Mapping rate** |
| --- | --- | --- | --- |
|  | CLL0275 | 44550832 | 32310360 (72.52%) |
|  | CLL0321 | 28957985 | 20536611 (70.92%) |
|  | CLL0335 | 41010026 | 29709952 (72.45%) |
|  | CLL0348 | 37145474 | 26477010 (71.28%) |
|  | CLL0377 | 47662223 | 36809394 (77.23%) |
|  | CLL0463 | 35330606 | 24319437 (68.83%) |
|  | CLL0515 | 37574191 | 25245656 (67.19%) |
|  | CLL0625 | 31513577 | 21397155 (67.90%) |
|  | CLL0659 | 21242110 | 15185296 (71.49%) |
|  | CLL0700 | 27901513 | 19871809 (71.22%) |
|  | CLL0775 | 36622012 | 25461659 (69.53%) |
|  | CLL0849 | 33769716 | 18055259 (53.47%) |
|  | CLL0949 | 52757088 | 18658224 (35.37%) |
|  | CLL6159 | 15834932 | 10659452 (67.32%) |
|  | CLL6536 | 9939151 | 5317563 (53.50%) |
|  | CLL8545 | 22550145 | 23275694 (70.00%) |
|  | CLL8919 | 24127512 | 16222071 (67.23%) |
|  | CLL9128 | 19981388 | 12626418 (63.19%) |
|  | CLL9130 | 25681002 | 17194768 (66.96%) |
| **Control Samples** | | | |
| 1. | SC1 | 28309650 | 19249263 (68.00%) |
| 2. | SC2 | 21560540 | 15266295 (70.81%) |
